# RNA sequencing reveals sexually dimorphic gene expression before gonadal differentiation in chicken and allows comprehensive annotation of the W-chromosome

**DOI:** 10.1186/gb-2013-14-3-r26

**Published:** 2013-03-25

**Authors:** Katie L Ayers, Nadia M Davidson, Diana Demiyah, Kelly N Roeszler, Frank Grützner, Andrew H Sinclair, Alicia Oshlack, Craig A Smith

**Affiliations:** 1 Murdoch Childrens Research Institute, Royal Childrens Hospital, Flemington Road, Parkville 3054 Melbourne, VIC, Australia; 2 Poultry Cooperative Research Centre, Armidale, NSW, Australia; 3 Department of Genetics, The University of Melbourne, Parkville, 3054, Melbourne, VIC, Australia; 4 Institute of Biological Sciences, Faculty of Science, University of Malaya, 50603, Kuala Lumpur, Malaysia; 5 The Robinson Institute, School of Molecular and Biomedical Science, University of Adelaide, 5005 Adelaide, SA, Australia; 6 Department of Paediatrics, The University of Melbourne, Flemington Road, Parkville, 3054 Melbourne, VIC, Australia

**Keywords:** Sex determination, Embryonic chicken gonad, W chromosome, Avian sex, RNA-seq

## Abstract

**Background:**

Birds have a ZZ male: ZW female sex chromosome system and while the Z-linked *DMRT1 *gene is necessary for testis development, the exact mechanism of sex determination in birds remains unsolved. This is partly due to the poor annotation of the W chromosome, which is speculated to carry a female determinant. Few genes have been mapped to the W and little is known of their expression.

**Results:**

We used RNA-seq to produce a comprehensive profile of gene expression in chicken blastoderms and embryonic gonads prior to sexual differentiation. We found robust sexually dimorphic gene expression in both tissues pre-dating gonadogenesis, including sex-linked and autosomal genes. This supports the hypothesis that sexual differentiation at the molecular level is at least partly cell autonomous in birds. Different sets of genes were sexually dimorphic in the two tissues, indicating that molecular sexual differentiation is tissue specific. Further analyses allowed the assembly of full-length transcripts for 26 W chromosome genes, providing a view of the W transcriptome in embryonic tissues. This is the first extensive analysis of W-linked genes and their expression profiles in early avian embryos.

**Conclusion:**

Sexual differentiation at the molecular level is established in chicken early in embryogenesis, before gonadal sex differentiation. We find that the W chromosome is more transcriptionally active than previously thought, expand the number of known genes to 26 and present complete coding sequences for these W genes. This includes two novel W-linked sequences and three small RNAs reassigned to the W from the Un_Random chromosome.

## Background

Sex determination in mammals and birds occurs at fertilization, with the differential inheritance of sex chromosomes [1]. Mammals have an XX/XY sex chromosome system, characterised by male heterogamety (XY), while birds have a ZZ/ZW system and female heterogamety (ZW). In eutherian mammals, almost all genes on the Y chromosome are associated with male development and fitness, namely the testis-determining *SRY *gene and genes required for spermatogenesis [2]. The Z and W sex chromosomes of birds evolved independently of the eutherian X and Y, and hence birds lack the *SRY *gene [3]. Like the mammalian X chromosome, the avian Z chromosome is large and gene-rich, while the W, like the mammalian Y, is typically small and largely heterochromatic. The 82 Mb chicken Z chromosome harbours over 1,000 genes and is highly conserved among avians [4,5], while the W has far fewer genes and appears to have undergone independent degradation across the various avian groups [6,7]. The exact roles of Z and W genes in avian sex determination remain to be fully resolved. Sex determination may involve a dominant-acting female determinant carried on the female-specific W chromosome, or a Z dosage-based mechanism could prevail [8]. Consistent with the latter, the Z-linked *DMRT1 *and *HEMOGEN *genes are involved in testis development in the chicken embryo [9,10]. However, a potential role for the W sex chromosome in avian sex determination cannot yet be excluded.

Attempts to address the potential role of the W chromosome in avian sex determination have been hampered by the poor understanding of this chromosome and its gene content. The chicken W chromosome has an estimated size of approximately 55 Mb, most of which (at least 70%) comprises repetitive elements of the *Xho*I, *EcoR*1 and *Ssp*1 classes [11,12]. In addition, long arrays of interstitial telomeric sequences have been described on the W. The exact number of genes on the W is obscure. The chicken genome, from a female Red Jungle fowl, was sequenced in 2004 [13] but assembly and annotation of the W has remained incomplete, and it is estimated that only 4% of the chicken W has been mapped [14]. This amounts to approximately 1.2 Mb of assembled sequence on the W in the most recent release of the Chicken genome (Gallus_gallus-4.0; November 2011) [15]. Until recently, around 12 bona fide genes had previously been verified as W-linked, including *CHD1W, ATP5A1W, HINTW, UBAP2W, NIPBL, hnRNPW, KCMF, SMAD2, SPIN, MIER3, ZFR *and *ZNF532 *[6,12,16-20] (chicken genome release 67, Ensembl) [21]. In a study of W chromosome gene expression in different breeds of chickens, Moghadam and colleagues recently increased the number of putative W genes to 21, with several genes suggested to have multiple copies [22]. However, expression data for these and other W genes at critical time points during embryonic sexual differentiation are lacking.

It has recently been proposed that avian sexual differentiation is at least partially cell autonomous. Cell autonomous sexual differentiation would involve purely intrinsic genetic factors, independent of any extrinsic signalling. In contrast, the long-held dogma is that vertebrate sexual differentiation is non-cell autonomous, that is, it does not rely purely on intrinsic factors in the cell and involves some degree of extrinsic signalling. (Sex hormones secreted from the gonads to induce female or male sexual dimorphisms, for example.) Zhao and colleagues (2010) used data from naturally occurring gynandomorphic chickens (birds that are half male and half female) and cross-sex embryonic tissue transplantations to show that sexual differentiation may be cell autonomous, at least partly independent of hormones [23]. This study would predict molecular sexual differentiation (that is, sexually dimorphic gene expression) prior to morphological sexual differentiation (that is, development of testes or ovaries and other sex dimorphisms) (reviewed in [24]). However, large-scale data in support of this hypothesis in avians have not previously been reported in any detail.

This study uses RNA sequencing to examine the degree of cell autonomous sexual differentiation in birds and to characterise the W transcriptome in chicken embryos. Comprehensive analysis of gene expression in early embryos reveals significant sexually dimorphic gene expression well prior to gonadal sex differentiation, involving both the sex chromosomes and autosomes. These findings support the idea that avian sexual differentiation is at least partly cell autonomous at the molecular level [23]. Different sets of genes showed sexual dimorphism in chicken blastoderms *versus *gonads, indicating that distinctive molecular pathways underlying sexual differentiation operate in different tissues. In addition, this analysis has allowed the full characterisation of gene expression from the enigmatic W (female) sex chromosome. Full characterisation of these W transcripts allows definitive comparisons with their Z homologues, shedding light on avian sex determination.

## Results

### Expression profiling of blastoderms and embryonic day 4.5 gonads reveals at least partial cell autonomous molecular sexual differentiation in chicken

Deep transcriptome sequencing was used to profile gene expression at two developmental time points in males and females; 12-h blastoderms (Hamburger and Hamilton stage 1) and day 4.5 embryonic gonads (stage 26) [25]. The rationale for using these times points was our focus on sex determination. Blastoderms represent the earliest accessible post-laying developmental stage, prior to primitive streak formation and gastrulation. This stage was chosen to specifically address the question of cell autonomous molecular sex differentiation pre-dating morphological differentiation. The second tissue, embryonic gonads at day 4.5, represents the time when the gonads are still morphologically identical in each sex ('bipotential').

Sequenced read-pairs were mapped to the chicken genome, (galGal3), using the TopHat 1.3.1 software [26]. The overlap of read-pairs with Ensembl genes was then counted. Differential expression analysis was undertaken by testing the female counts against male counts at both time-points using edgeR [27], with a false discovery rate (FDR) <0.05. Genes known to be expressed sexually dimorphically in E4.5 gonads served as positive controls. For example, *DMRT1 *and *AMH *are known to be male upregulated by approximately two-fold in E4.5 gonads, and *FOXL2 *is expressed only in female gonads at E5.0. Meanwhile, both *Aromatase *and *SOX9 *are expressed after E4.5, and were expected to be non-dimorphic in our datasets [28]. These patterns were confirmed in the RNA-seq (see Additional file 1, Figure S1), validating the sequencing results.

**Figure 1:**
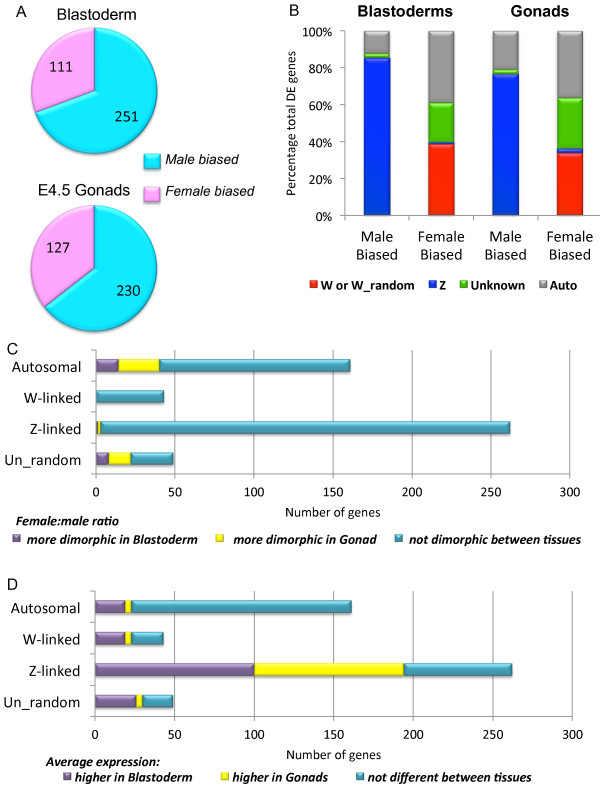
**RNA-seq analysis of embryonic chicken blastoderms and embryonic day 4.5 (stage 26) gonads**. (**A**) Differentially expressed annotated genes (Ensembl-based). Number of genes showing either male-biased differential expression (FDR <0.05; blue) or female-biased expression (FDR <0.05; red) in blastoderms and E4.5 gonads. (B) Chromosomal allocation of differentially expressed genes, based on annotated gene data. In blastoderms, female biased genes were located on the W or W_random chromosome (red), the autosomes (grey), or the un-assembled Un_random chromosomes (green). One Z-linked gene showed female-biased expression (aqua). The vast majority of male-biased genes were Z-linked (aqua), and autosomal (grey). Two were on Un_random chromosomes and zero on the W chromosome. A similar pattern was observed in the gonads, with the majority being Z-linked in males, and W-linked or on the Un_random in females. (**C**) Bar graph illustrating the number of genes with sexually dimorphic expression in at least one tissue on the W, Z, autosomal and Un_random chromosomes. Genes were tested for different patterns of sexually dimorphic expression between tissues, and are grouped as to whether they show a significantly larger female: male ratio difference in the blastoderms (purple), the gonads (yellow) or whether no significant difference is observed (aqua) (FDR <0.05) (See also Additional file 4, Table S1c). (**D**) Bar graph of genes of genes that are significantly sexually dimorphic in at least one tissue on the W, Z, autosomal and Un_random chromosomes, similar to Fig. 1C). Here, genes are grouped based on a change in average expression between tissues. Shown are the number of genes which are significantly more highly expressed in the blastoderms (purple), higher in the gonads (yellow), and no significant change (blue) (FDR <0.05) (See also Additional file 4, Table S1c).

Our annotation-based differential expression analysis [29] revealed hundreds of genes differentially expressed between males and females in both tissues (362 in blastoderms and 357 in the gonads) (Figure 1A, and Additional file 2 and 3). This indicated robust sexually dimorphic gene expression pre-dating gonadal development, and supports the notion of cell autonomous sexual differentiation at the molecular level in chicken. In the blastoderm, most of the genes upregulated in males were Z-linked (85%), with a smaller but significant proportion annotated to autosomes (12%) (Figure 1B). This indicated that the Z chromosome is not fully dosage compensated, with the mean expression of Z-linked genes 1.6-fold higher in males compared with females (see Additional file 1 Figure S2), as reported previously [30-32]. Meanwhile genes upregulated in females were annotated to the W chromosome (38%), autosomes (39%), or to the Un_random chromosome (21%) (Figure 1B). The latter represents a virtual chromosome of un-assembled and un-localised chicken sequence. A similar trend was observed in the E4.5 gonads (Figure 1B). Notably, a very small number Z linked sequences were female-biased, in both tissues (Figure 1B). These derived from the *MHM *locus (Male Hypermethylated), a curious sequence that has previously been reported to be female specific and hypothesised to play a role in localised dosage compensation (upregulation of some neighbouring Z genes in females) [33,34].

Some genes involved in sexual differentiation of the gonads begin to be dimorphically expressed between the sexes only in the gonads (for example, FOXL2). These genes therefore showed a different pattern of sexual dimorphism from blastoderm to gonad; that is, the female: male ratio was significantly different between tissues. To identify additional genes showing this difference in sexual dimorphism, we used a robust statistical test for the difference in female: male fold change between tissues (see Additional file 1 and 4). Of all of the 43 W-linked Ensembl sequences we detected, none of these genes showed a different pattern of sexual dimorphism (Figure 1C). For Z-genes, of which 262 were differentially expressed in at least one tissue, only three genes (1%) showed a significantly different pattern of sexual dimorphism between tissues (Figure 1C). This included two MHM locus genes that show a large increase in female expression in the gonads, and the Endothelial Tyrosine Kinase (*TEK*) gene that was more dimorphic in blastoderm (ENSGALG00000018479, ENSGALG00000023324, ENSGALG00000001840). However, 40 autosomal genes (25%) and 22 Un_random genes (45%) showed a different pattern of sexual dimorphism between tissues (Figure 1C). These data indicate that sex-specific molecular pathways that manifest in the blastoderms differ to those in gonads, primarily due to difference in autosomal gene expression. Given that sex chromosome genes did not generally deviate in their female: male ratio between the two tissues, it is interestingly to speculate how these genes can then activate different downstream genes in blastoderms and gonads. In the case of *DMRT1*, a known testis determinant that is differentially expressed with a similar ratio in both tissues, we found that the average expression level ((male+female)/2) of this gene dramatically increased from blastoderm to gonad. We therefore tested all genes for significant differential expression between tissues, regardless of sex (see Additional files 1 and 4). Indeed, a number of sexually dimorphically expressed W and Z genes also showed differential expression between the tissues, that is, W-genes - 23 (53%), Z-genes - 194 (74%) (Figure 1D and Additional file 4). Those genes upregulated in the gonads (Figure 1D and Additional file 4) are therefore interesting candidate gonadal sexual differentiation genes. Altogether, the data indicate that the relative expression levels of dimorphic sex-linked genes could explain their ability to regulate different downstream genes in different tissues.

To shed light on the molecular pathways that might underlie cell-autonomous sexual differentiation, we first screened the datasets for genes implicated in gonadal sex differentiation. A list was compiled of 117 genes previously linked tovertebrategonadogenesis (see Additional file 5). The set of genes differentially expressed between male and female blastoderms was significantly enriched with these gonadogenesis genes (*P *= 0.0098, Fisher's exact test), mostly due to non-compensated Z-linked genes (for example, *17βHSDB4 *(ENSGAL00000002187), *DMRT1 *(ENSGAL00000010160) and *CFC1B *(ENSGAL00000012623)). Only one differentially expressed autosomal 'implicated gonadal gene' was found differentially expressed in this tissue (*VNN1*, ENSGALG00000013992) (Additional file 2). No genes previously proven to have a role in gonadal sex differentiation were found among the W-linked sequences. Taken together, these data indicate that sex chromosome genes do not activate known sexual differentiation pathways in blastoderms of either sex.

In contrast to the blastoderms, the gonads showed a different set of differentially expressed autosomal genes, many of which have a known link to gonadal sex differentiation, such as *FOXL2, Anti-Müllerian Hormone (AMH*), *INHA (Inhibin-A*) and *HSP70 *(ENSGALG00000011715). This indicates that the sex chromosomes have initiated developmental programs specifically associated with gonadal sex differentiation at embryonic day 4.5 (stage 25), 1 to 2 days prior to the onset of gonadal sexual differentiation (stages 29-30).

These findings show that different sexually dimorphic molecular pathways are engaged by the sex chromosomes in the two different tissues examined here. However, most of the differentially expressed genes detected in blastoderms and gonads had no previous connection to sexual differentiation. To characterise potential pathways activated in female *versus *male tissues, we assessed gene ontology of all genes that were exclusively differentially expressed in only blastoderms or gonads, using the DAVID programs [35]. The top three clusters of GO terms for each group are shown in (Additional file 1, Figure S10). Given the low number of genes, very few GO terms showed significant enrichment when we corrected for multiple testing (Benjamini), however numerous genes involved in cell stress and DNA damage repair showed sex differential expression in the blastoderm. Various members of the hepatic fibrosis pathway were also differentially expressed (*A2M *and *Col3A1 *upregulated in females, and *IL1R2 *and *EGF *in males). Sex-specific gene expression in the liver has been previously described, involving >1,000 genes that affect a wide range of biological processes [36]. For genes specifically differentially expressed in the gonads, the top GO terms included neuroactive ligand-receptor interaction (*P *value = 0.0081). These genes include the GABA receptor alpha 4 and two glutamate receptors. Among the list of autosomal genes expressed in the day 4.5 gonads in a sexually dimorphic fashion were several transcription factors not previously linked to sex *per se *(four upregulated in females and six in males) (Additional file 2). These data reveal the engagement of different molecular pathways at the two tissues assayed.

As mentioned previously, Z- and W-linked genes generally showed similar dimorphic expression in both blastoderms and gonads (Figure 1C), while many autosomal genes were only differentially expressed in one tissue (Figure 1C). In addition, a significant proportion of genes annotated to the Un_Random chromosome showed a stable dimorphic expression profile (Figure 1C). These unassigned sequences may in fact map to sex chromosomes, especially the poorly annotated W chromosome. Evidence for this conclusion was supported by their sex expression ratios, which were similar to those of the sex chromosome genes (Additional files 2 and 3). As the chicken W sex chromosome and its potential role in avian sex determination is currently poorly understood, we exploited the RNA-seq data to definitively annotate these sequences and to address W-linked gene expression.

### Annotation of the W chromosome

Given the robust W-linked gene expression in both blastoderms to E4.5 gonads, we considered that this chromosome might play an important role in sex determination and cell autonomous molecular sex differentiation. However, the chicken W sex chromosome is currently incompletely annotated and its sequence is only partially assembled. In light of the challenges associated with sequencing the W chromosome, we investigated the W transcriptome and its potential role in avian sex determination in more detail. Two approaches were used to construct full-length open reading frames for the W transcriptome, genome-guided and genome independent (*de novo*) assembly.

Potential mis-annotated W genes were initially identified by their female specific expression (Ensembl) (see Methods and Materials). However, a significant fraction of reads also mapped outside annotated genes. Most notably, 62% of reads that mapped to the Un_random chromosome were not in annotated genes (Additional file 1, Figure S3). We hypothesised that some of these sequences were unannotated W-linked contigs. In order to identify unannotated genes, we extended our analysis of the RNA-seq data using a genome guided transcript assembly procedure, Cufflinks [37]. The Cufflinks analysis was performed by mapping to the chicken genome (Galgal4), and a set of chicken transcripts was created by running Cufflinks 1.3.0 on the mapped reads (see Additional file 1). A significant proportion of expressed W genes identified through the Cufflinks analysis encoded retroviral elements with at least one open reading frame that showed homology to a retroviral protein (with an e-value <0.001, using BLAST). Together with pseudogenes, these sequences were filtered out of subsequent analysis (see Methods and Materials and Supplementary Methods in Additional file 1).

This analysis allowed the compilation of 26 W-linked genes (Table 1). Most of these genes have previously been suggested or confirmed to be W-linked [22], but we identified two novel W-linked sequences, *TXN-like *and *SUB1-W*. In addition, two W-linked genes, *RPL17-*W and *HNRPK-W*, lie on the Un_random chromosome as described in [22] and confirmed by us. Located within the intronic region of these genes are two annotated small nucleolar RNAs (*SNORD58-W-1 *and *SNORD58-W 2*) and one microRNA (*mIR-7b-W*) that have not been documented to be W-linked. Give the location of the host gene and the presence of a gametologue on the Z chromosome, these genes should be reassigned to the W chromosome.

**Table 1:** Annotation and expression of chicken W chromosome genes.

Gene	Description	Genomic Location	Expression (FPKM) Female		% ID with paralog		dN/dS
			Blastoderms	E4.5 Gonads	DNA	Protein	
*ATP5A1-W*	ATP synthase subunit alpha	ChrW W_random	101	155	89.3	95.4	0.280

*BTF3-W*	Transcription factor BTF3-like	Autosome	86.3	141	90.9	94.4	0.160

*C18ORF25-W*	Uncharacterized protein C18ORF25	ChrW W_random ChrUn	0.874	3.93	90.3	83.7	0.434

*CHD1-W*	Chromodomain helicase DNA binding protein	ChrW W_random autosomal	6.81	9.35	86.8	88.1	0.126

*FAF *	Female Associated Factor	ChrW W_random	15	21.2			

*GOLPH3-W*	Golgi phosphoprotein 3-like	ChrUn	2.82	24.9	92.7	95.6	0.080

*HINT-W*	Histidine triad nucleotide binding protein	ChrW W_random	1780	1170	41.0	48.5	0.608

*HNRPK-W*	Heterogeneous nuclear ribonucleoprotein K	ChrUn	103	100	94.2	99.3	0.040

*KCMF1-W*	E3 ubiquitin-protein ligase KCMF1-like	ChrW W_random	10.1	21.6	92.0	93.2	0.181

*MIER3-W*	Mesoderm induction early response 3	ChrUn	7.42	19.9	92.9	92.5	0.265

*NEDD4-like-W*	Neural precursor cell expressed developmentally downregulated 4-like	ChrUn	0.048	1.92	82.4	83.0	0.209

*NIPBL-W/SCC2-W*	Nipped B/sister chromatid cohesion 2	ChrW W_random	7.78	7.39	89.9	90.0	0.148

*RASA1-W*	Ras GTPase-activating protein 1-like	ChrW W_random ChrUn	2.77	10.5	81.4	89.4	0.132

*RPL17-W*	Ribosomal protein 17	ChrUn	368	332	96.7	100.0	0.000

*SMAD2-W*	Mothers against decapentaplegic homolog 2-like	ChrW W_random	19.2	30	92.4	96.4	0.024

*SMAD7-W*	TGF-beta signal pathway antagonist Smad7	ChrUn	4.47	4.95	93.8	93.6	0.208

*SPIN-W*	Spindlin	ChrW W_random	1.8	10.6	95.7	98.5	0.045

*ST8SIA3-W*	Sia-alpha-2,3-Gal-beta-1,4-GlcNAc-R: alpha 2,8-sialyltransferase-like	ChrW W_random	4.31	3.16	94.7	95.7	0.105

*SUB1-W*	Activated RNA polymerase II transcriptional coactivator p15-like	ChrUn	11.6	26.6	94.5	96.0	0.097

*TXN-like1-W*	thioredoxin	ChrUn	7.16	19.6	94.5	95.2	0.144

*UBAP2-W*	Ubiquitin associated protein 2	ChrW W_random	16.3	11.9	92.1	89.6	0.263

*UBE2R2-W*	Ubiquitin-conjugating enzyme E2R 2	ChrW W_random	5.38	17.4	94.4	100.0	0.000

*VCP-like-W*	Valosin containing protein	ChrUn	64.4	128	95.5	99.9	0.003

*ZFR-W*	Zinc finger RNA-binding protein	Autosome ChrUn	4.47	7.51	93.8	94.1	0.172

*ZNF532-W*	Zinc finger protein 532-like	autosome	3.52	5.59	94.0	94.0	0.181

*ZSWIM6-W*	Zinc finger SWIM domain-containing protein 6-like	ChrW W_random autosome ChrUn	0.54	4.73	91.7	92.4	0.172

*Mir-7b-W*	MicroRNA 7b (in intron of HNRPK-W)	ChrUn	unknown	unknown	93.6		

*SNORD58-W 1*	Small nucleolar RNA SNORD58 (in intron of RPL17-W)	ChrUn	unknown	unknown	97.0		

*SNORD58-W 2*	Small nucleolar RNA SNORD58 (in intron of RPL17-W)	ChrUn	unknown	unknown	89.7		

*SNORD121A-W 1*	Small nucleolar RNA SNORD121A (in intron of UBAP2-W)	ChrW W_random	unknown	unknown	90.6		

*SNORD121A-W 2*	Small nucleolar RNA SNORD121A (in intron of UBAP2-W)	ChrW W_random	unknown	unknown	90.4		

A list of the chicken W genes. *Genomic Location *gives the chromosome/s where the gene sequences can be found in the chicken reference genome (galGal4). *Expression *shows the average expression levels as an FPKM value (fragments per kilobase of exon per million fragments mapped) for females from the blastoderm and gonad (E4.5) tissue. The % ID gives the percentage identity of the W gene's open read frame against its Z gametologue for both DNA and amino acid sequences. dN/dS is the ratio of synonomous to non-synonomous substitutions, also across the open read frame of the W genes and their Z gametologues.

**Figure 2:**
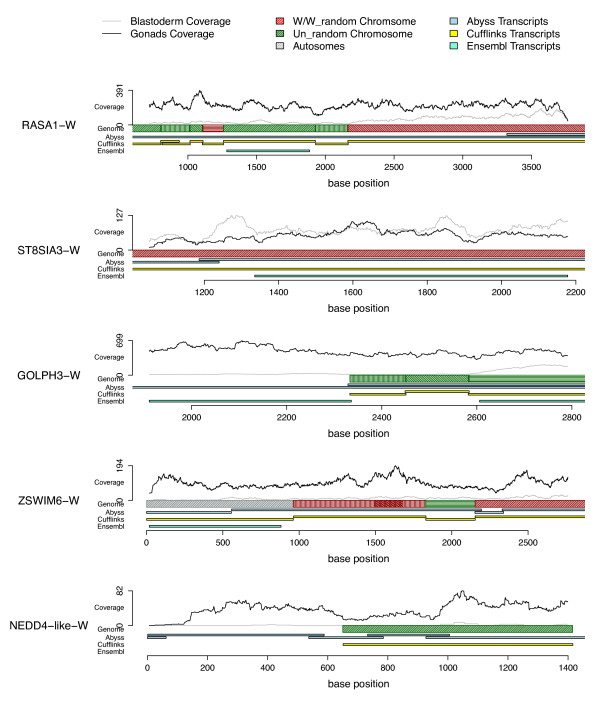
**Delineation of complete W-linked transcript sequence**. Complete transcript sequences for all W-expressed genes were determined using a combined approach of assembling transcripts from Cufflinks, the Abyss *de-novo *assembly and the latest chicken annotation data (Ensembl). An example of the open reading frame for five W-linked genes is shown. The hatched rectangles represent the different genomic regions to which sequences could be aligned: the W/W_random chromosome (red), Un_random chromosome (green), autosomes (grey) and gaps represent absent genomic sequence. The coloured bars below show the corresponding transcripts defined by Ensembl (aqua), Cufflinks (yellow) and ABySS (blue) analyses. The plots along the top represent the read coverage for the female gonadal sample (black) and the blastoderm sample (grey).

During both the Ensembl and Cufflinks analyses, it was noted that in at least 12 cases, multiple identified transcripts encoded the same putative protein (Suppl. Table 3). While in some cases, such as *HINT-W *and *FAF*, this was due to multiple copies of the gene in the genome, in many cases there was a single copy of the gene, but it had been split across non-contiguous or gapped regions of the genome. Genome-guided assemblies such as Cufflinks are limited by the quality of the genome and transcripts cannot be assembled across segments of the genome which are not correctly scaffolded or which contain gaps in the assembled sequence. This is particularly true for the Un_random and W_random chromosomes, which contain unassembled fragments of the genome. To address this issue we performed a genome independent (*de novo*) transcript assembly using the ABySS software [38,39] and used the *de-novo *assembled transcripts to reassemble different Cufflinks genes together. This enabled genes previously assigned to different regions of a chromosome or even across different chromosomes to be joined together into a single gene (see Additional file 1 for details). Figure 2 shows the results of such analysis for five representative genes, *RASA-W, ST8SIA-W, GOLPH-3-W, ZSWIM6-W *and *NEDD4-W *(the remaining assembled W transcripts are given in Figure S4; see Additional file 1). Deduced complete transcripts are shown together with the sequences annotated by Ensembl and those derived from Cufflinks and ABySS assemblies. For example, the *RASA-W *transcript was assembled by joining seven sequences previously assigned partly to the W and partly to various fragments of the Un_random and W_random chromosome.

Using this approach, the open reading frames of W-linked mRNAs could be completely assembled, with the exception of one gene, *NEDD4-like*-W. This resulted in the characterisation of ten genes where previously only part of their ORF was known, due to the poor assembly of the W chromosome. Of these genes, we estimate that, on average, 60% of open reading frame sequence was previously missing from that available in either Ensembl or on Genbank (Additional file 6). Subsequent expression analysis of W genes was carried out by mapping reads to the newly assembled complete cDNA sequences. Final FPKM values (Fragments Per Kilobase of exon per Million fragments mapped) are presented in Table 1.

The complete W-linked transcripts identified in this RNA-seq screen showed gene ontologies with varying functions, none of which are patently associated with sexual differentiation (Table 1 and Table S3 - Additional file 6). However, the lists included genes specifying proteins associated with transcriptional regulation (*ZSWIM6-W, ZNF532-W, MIER3-W, BTF3-W, SUB1-W*), signalling (*SMAD2-W, SMAD7-W, RASA1-W*), the ubiquitination pathway (*UBAP2-W, UBE2R2-W*), the antioxidant thioredoxin (*TXN-like-W*), the ATP synthase, *ATP5A1-W*, and the aberrant nucleotide-binding protein, *HINTW*. As noted above, most W-linked genes did not show significant expression changes between the two tissues examined (Table 1). The most highly expressed genes across both tissues were *HINT-W, RPL7-W, ATPA5A1-W, BTF3-W, VCP-like-W *and *hnRNKP*.

### Confirmation of female-restricted expression and W-linkage

**Figure 3:**
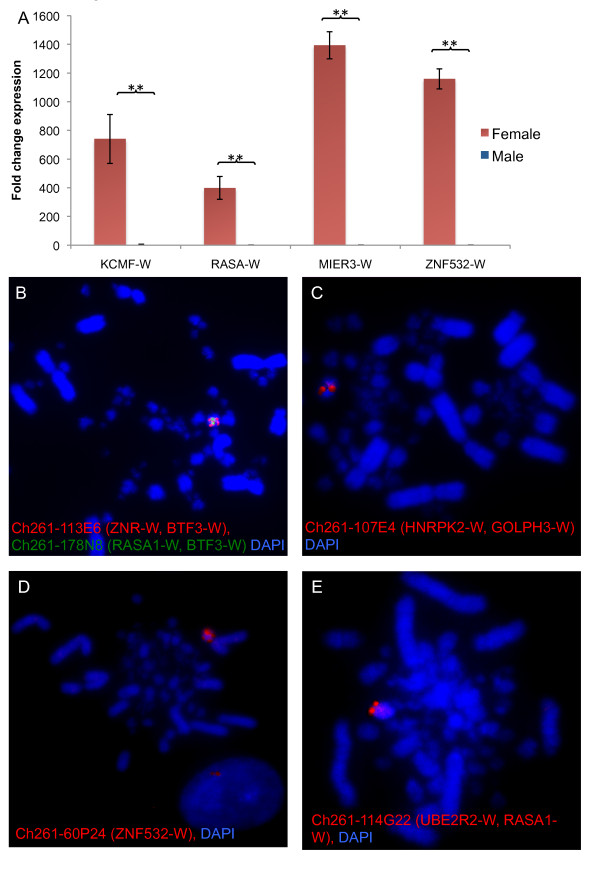
**Validation of RNA-seq by quantitative RT-PCR and Confirmation of W-linkage**. (**A**) Blastoderm expression analysis of four representative W genes, *KCMF-W, RASA-W, MIER3-W *and *ZNF532*. Expression was detectable in females (red) but not in males (blue). Normalised W gene expression is shown; mean +/- SEM; *n *= 3; ** *P *<0.05. (**B-E**) FISH mapping of genes identified by RNA-seq to the W sex chromosome in female chicken metaphase spreads. BAC clones were used as probes. (B) BAC clone Ch261-113E6 (*ZNR-W, BTF3-W*) (red) and BAC Ch261-178N8 (*RASA1-W, BTF3-W*) (green). (**C**) BAC clone Ch261-107E4 (*HNRPK2-W, GOLPH3-W *(red). (**D**) BAC clone Ch261-60P24 (*ZNF532-W, SnoR58-W*) (red). (**E**) BAC clone Ch261-114G22 (*UBE2R2-W, RASA1-W, SnoR121A-W*) (red). Metaphase chromosomes are stained with DAPI (blue). A single signal was detected in each case and only in female cells, confirming W linkage.

The RNA-seq data demonstrated that the chicken W sex chromosome harbours more genes than previously thought and that these genes show robust transcriptional activity. All W genes were expressed in both blastoderms and E4.5 gonads. To validate the RNA-seq, quantitative RT-PCR was carried out on four representative genes, using W-gene specific primers. PCR amplification was detected in female but not male blastoderm and gonadal RNA samples (Figure 3A and 3B), thus confirming female specific expression. For some of these genes, whole mount *in-situ *hybridisation also confirmed female gonad-specific expression (Additional file 1, Figure S5). FISH mapping was used to validate W linkage, showing a single signal in female but not male cells (Figure 3B-E and Additional file 1, Figure S6). In addition, for predicted or novel genes not yet assigned to the W, PCR analysis confirmed that they are present specifically in female but not male chicken genomic DNA (see Materials and Methods and Figure S6 in Additional file 1).

### Conservation and relative expression of W-linked genes and their Z gametologues

The complete W-linked transcripts assembled in this study were used to screen for homologues on the Z chromosome (gametologues). All W-linked genes with the exception of *FAF *(Female Associated Factor) were found to have gametologues on the Z. There was high sequence homology between almost all W- and Z-linked copies at both the DNA level (average of 88.4% identity, Table 1) and the protein level (average of 90.3% identity, Table 1). An exception was the gene *HINTW*, which showed 41% sequence and 48.5% amino acid homology with its Z gametologue (Table 1). Evolution of novel function among W-linked genes was examined by calculating the rates of synonymous and non-synonymous substitution for each Z-W gene pair [40] and is represented in Table 1, and as a sliding window across each gene pair in Figure S7 (Additional file 1). The dN/dS value for *HINTW *was the highest of all W genes (0.6) indicating that it has undergone the least amount of purifying selective pressure.

**Figure 4:**
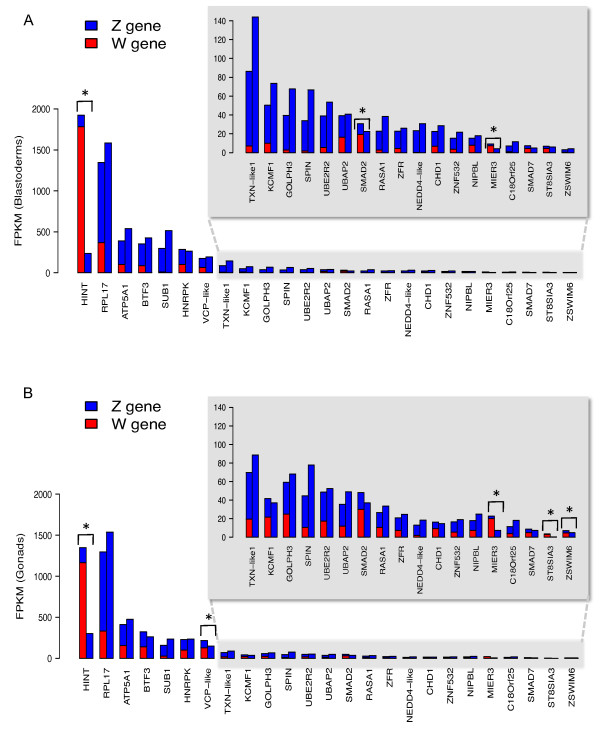
**Expression levels of W/Z gametologue pairs**. Expression of W-linked genes (red) compared to their Z-linked gametologues (blue), for blastoderms (**A**) and gonads (**B**). The total combined expression of gametologue pairs is shown for females (red - W/blue - Z, left bar in pair) and males (ZZ -blue only, right bar in pair). The shaded data are shown on an adjusted FPKM scale (inset). Genes with significantly different expression between the sexes are identified (* *P *<0.01).

The combined expression of the W and Z gametologues was assessed in both blastoderms and gonads and is shown in Figure 4. For virtually all expressed W-linked genes, the Z gametologue was also expressed, and in both tissues. (The exception was *FAF*, which lacks a Z gametologue). Total expression from the W and Z gametologues in females was in most cases comparable to the expression of the two Z-linked copies in males, where typically the Z and W contributed equally to the total expression in females (Figure 4A, B). This suggests that most W/Z gametologues in the chicken embryo effectively operate in an autosomal-like fashion (having two functional copies in both male and female). However in some cases, the combined W/Z gene expression in females was significantly higher than the total Z-linked expression in males and was primarily due to W transcription. In the blastoderm, this was the case for *HINT, SMAD2 *and *MIER-3*(Figure 4A). In E4.5 gonads, female expression was higher for *HINT, MIER3*, the putative transcription factor Z*SWIM6, VCP-like *(Valosin-containing protein) and *ST8SIA3 *(a sialyltransferase-like gene) (Figure 4B).

## Discussion

This study indicates that cells of the chicken embryo commence sexual differentiation at the molecular level early in development, through sexually dimorphic gene expression from both sex chromosomes and autosomes. This involves the enigmatic W sex chromosome, and it supports recent observations indicating that sexual differentiation may be at least partly cell autonomous in birds. This does not, however, exclude an important role for non-cell autonomous sexual differentiation, which involves hormonal signalling and is clearly very important in birds as it is in all animals. However, the data presented here support the notion that cell autonomous factors are also important, and there is very likely an interaction between both mechanisms to ensure complete and proper phenotypic sexual differentiation.

Previous studies have also reported sexually dimorphic gene expression in chicken embryos pre-dating gonadal development [32,41,42]. These studies utilised microarrays or subtractive hybridisation to uncover genes regulated in a sex-specific manner prior to gonadal differentiation in the chicken embryo. Our study utilised the earliest time point to date (12 h, stage 1 blastoderms), and has employed the most sensitive and comprehensive method available to assay gene expression. RNA-seq detects all expressed genes, including those that are unannotated, compared to microarray analysis, which only detects sequences arrayed on the microarray [29]. The finding that sexually dimorphic gene expression can pre-date gonadal sex differentiation is not limited to birds, but is likely to be a feature of all animals. Sex differences in gene expression before gonadogenesis have been reported in mouse and bovine embryos [43,44].

We found that W- and Z-linked genes showing sexually dimorphic expression in blastoderms largely maintained this differential expression profile in the gonads, pointing to a pervasive role for the sex chromosomes in the continuity of cell autonomous sexual development. The sexually dimorphic expression of autosomal genes indicates that female- and male-specific pathways are engaged as early as the blastoderm stage. However, a different set of autosomal genes showed sexually dimorphic expression in the undifferentiated gonads, most notably genes implicated in gonadal development. This indicates that the sex chromosomes activate different sex-specific pathways in different tissues.

The mechanism through which the Z and W sex chromosomes act to direct sexual differentiation pathways in early embryos is still unclear. One hypothesis is that the W chromosome carries a dominant-acting female determinant. This could confer 'femaleness' in a cell autonomous fashion early in development, but it may also initiate ovarian differentiation of the gonads at day 4.5. The RNA-seq data presented here has allowed us to draw some conclusions regarding the potential role of the W in avian sex determination. Contrary to previous assumptions, we found that the W is highly transcriptionally active in chicken embryos. Our novel analysis strategy allowed us to construct full-length transcripts for essentially the entire W transcriptome in two tissues. Using these we could correct and expand the annotation of the chicken W, providing complete transcript sequences and open reading frames for almost all 26 genes (Table 1 and Table S3 - Additional file 6). Most of these genes have previously been reported as W-linked in chicken [20,22]. However, a thorough assessment of expression prior to or during gonadal development has been lacking, and limited sequence analysis has been carried out for these W-linked genes. In addition, we describe here two additional W linked genes and three W-linked small RNAs (Table 1). The assembly of full-length W transcripts described has allowed the accurate comparison of W and Z gametologues, providing key information on the degree of divergence between gene pairs and their relative expression levels. Most of the expressed W genes have open reading frames that are almost or completely homologous to their Z-linked gametologues (Additional file 1, Figure S7), which were also expressed in both blastoderms and gonads. This includes both the known and novel genes identified here. These W and Z genes are therefore likely to be acting as '*de facto *autosomal' pairs, and hence almost all W-linked genes are unlikely female sex determinants. Indeed most of these genes encode proteins associated with general functions in the cell, such as ubiquitination, lipid and energy metabolism, and chromosome structure (Table 1). The two exceptions are *HINTW*, which is divergent from its Z gametologue, and *FAF*, which lacks a Z gametologue.

*HINTW *(Histidine Triad nucleotide binding, W-linked) is highly expressed in female chicken embryos at the blastoderm and gonadal stages (Table 1) [17]. The high level of *HINTW *expression in females most likely reflects the multiple copies of *HINTW *that reside on the chicken W [17]. While an endogenous HINTW protein has yet to be demonstrated, it is predicted to specify an aberrant nucleotide binding protein, lacking a key catalytic domain. The Z-linked copy, *HINTZ*, encodes a bona fide nucleotide binding protein, mediating the hydrolysis of nucleoside monophosphates [17] and it is hypothesised that HINTW may operate in a dominant negative fashion, blocking the function of HINTZ [8,45]. While experimental over-expression of *HINTW *mRNA fails to alter male development in chicken embryos [46], the potential role of *HINTW *in avian sex determination requires closer examination. One W-linked transcript detected in our screen did not have a Z gametologue; *FAF *(*F*emale *A*ssociated *F*actor) [23]. The *FAF *gene product shows no clear homology to known proteins and its potential role in sexual differentiation is not clear at this stage.

The same set of W-linked genes expressed in female blastoderms was also expressed in E4.5 female gonads, yet a different set of autosomal genes was expressed. Therefore, if W-linked genes play a role in female development, they must engage different pathways in the different tissues. In the developing female gonad, a putative ovary determinant would directly or indirectly activate expression of the key *FOXL2 *gene, which is thought to then activate *CYP19A1 *and hence the oestrogen synthesis that is central to ovarian differentiation. Since we detected female enriched *FOXL2 *expression already in E4.5 gonads, the direct activator of this gene must also be present in our E4.5 female dataset (Additional file 5). This requires further analysis.

The alternative mechanism mediating avian sex determination involves Z-gene dosage. As there is no global Z chromosome dosage compensation system in birds, a sex determining mechanism may rely upon Z gene dosage inequality between the sexes. One or more non-dosage compensated Z-linked genes could direct sexual differentiation in individual cells. Our findings indicate that like the W, many Z-linked genes show sexually dimorphic expression that does not differ between the tissues assayed, with only 1% showing significantly different levels of dimorphic expression between the two tissues. This indicates that the sex chromosome influence sexual differentiation in cells, but this does not explain how sex-linked genes could then trigger sexually dimorphic expression of different autosomal genes in each tissue. Many Z- and W- linked genes showed different average expression levels between tissues (Figure 1D), and therefore while female: male ratios may not differ, changes in total expression levels may play a role in each tissue. For example, the Z-linked testis-associated genes, *DMRT1 *and *HEMOGEN*, are more highly expressed in the gonads compared to the blastoderms. Therefore it is possible that additional Z-linked genes may play a role in sexual differentiation in different tissues through changes in their relative expression. It is noteworthy in this regard that many Z-linked genes with sexually dimorphic expression in the gonads were sex-related (Additional file 5). Previous studies have reported a clear bias for genes associated with sex and reproduction located on the avian Z sex chromosome [47,48], making sex determination via Z dosage and expression levels an attractive model.

It has recently been hypothesised that multiple parallel pathways leading to sexual differentiation probably exist in mammalian embryos [49]. Similarly, in birds, molecular pathways controlling sexual differentiation in a cell autonomous fashion in the early embryo would be different to those operating specifically to induce ovarian *versus *testicular development at later stages. This idea is supported by our findings that different sets of autosomal genes show sexually dimorphic expression in the blastoderm compared to the E4.5 embryonic gonad. It is noted, however, that ultimately, proper sexual differentiation must involve the interaction of both cell autonomous and non-cell autonomous factors. Despite the advances brought by this research, the exact mechanism underlying avian sex determination is still unclear.

## Conclusions

The data reported here show sexually dimorphic gene expression in chicken blastoderms and gonads prior to gonadal sex differentiation. This supports the notion that sexual differentiation begins at the molecular level in a cell autonomous manner in the chicken embryo. This involves both sex-linked and autosomal genes, with different sets of genes being expressed in the two tissues, demonstrating the engagement of different sexually dimorphic pathways. While sexually dimorphic gene expression from the Z and W sex chromosomes does not dramatically differ between developmental stages, we show that significant differences in average levels of expression of sex-linked genes may be responsible for activation of different signalling pathways in a tissue specific manner. Furthermore, we comprehensively define the W chromosome transcriptome for two early embryonic tissues, annotating W genes and providing complete open reading frames for most genes, and characterising two novel W-linked genes and several small RNAs.

## Materials and methods

### Tissue collection

All experiments were carried out on a single line of Specific Pathogen Free (SPF) embryos (SPAFAS) from the SPF White Leghorn strain of chick (Lohman-LSL).

*Blastoderms*: Eggs were incubated for 12 h at 38°C and the blastoderms were dissected in cold DEPC-treated PBS. Hamburger and Hamilton (HH) stage 1 blastoderms were taken, and any showing developed primitive streaks were discarded. Only the area pellucida was dissected from the vitelline membrane and used, and any remaining yolk was removed. A small piece of blastoderm was taken for genetic sexing by PCR (see below), and the remainder of the blastoderm was stored at -80°C until RNA extraction was performed.

*Gonads*: SPF eggs were incubated until stage 26 (embryonic day 4.5). Paired gonads were removed and stored at -80°C. Handplate tissue was used for sexing. Embryos were sexed by PCR as described previously [50,51].

### RNA extraction and sequencing

Tissues were pooled according to sex. Twelve blastoderms or 16 paired gonads were pooled for each replicate (two male replicates and two female replicates were collected for each tissue). Total RNA was extracted using the RNeasy micro kit (QIAGEN) (which enriches for mRNAs, that is, RNAs >200 base pairs in length). On column DNAsing was performed to remove contaminating genomic DNA. The resulting RNA was poly A-selected, reverse transcribed, fragmented, bar-coded and sequenced using the Illumina HiSeq2000 at Australian Genome Research Facility (AGRF) in Melbourne. We sequenced 100 base pair, paired ends reads. Four lanes were used and all samples were bar-coded and run on each lane. The RNA was sequenced to a depth of approximately 20 million read-pairs per sample per lane, giving a total for each sample of 80 million read-pairs (160 million reads). These data are available on the NCBI Sequence Read Archive (accession number SRA055442).

### RNA-seq analysis

Sequences were screened to remove poor quality bases, and subsequent analysis of the data followed the three methods shown in Additional file 1, Figure S8 and described in detail in the Supplementary Methods (Additional file 1). Briefly, we mapped the trimmed reads to the genome and counted reads which overlapped Ensembl genes, we performed genome-guided transcriptome discovery using Cufflinks [52] and we assembled the transcriptome independent of the chicken genome using ABySS [38][39]. For the Ensembl differential expression analysis and W-linked gene identification we mapped the read-pairs to the chicken genome, galGal3, using the TopHat 1.3.1 software [26], with default settings. We counted reads which mapped to annotated genes giving a count for each gene in each sample and then used edgeR for differential expression (DE) analysis. edgeR uses a Generalized Linear Modeling (GLM) framework to model the gene counts as a negative binomial distribution. Variation estimates are stabilized by borrowing information between genes using an empirical Bayes approach [27] and hypothesis tests are performed using a likelihood ratio test. This method takes into account both biological and technical sources of variation even for small numbers of replicates. In this case we had 3 degrees of freedom to estimate biological variability [53]. Further details are described in the Supplementary Methods (Additional file 1). Differential expression for the Cufflinks defined genes was performed in a similar manner to the Ensembl analysis, however we mapped to the updated chicken genome Galgal4 and the set of chicken transcripts was created by running Cufflinks 1.3.0 on the mapped reads (see Additional file 1 - Supplementary Methods).

#### *De-novo *W gene assembly

The *de-novo *transcriptome assembly was performed using Abyss version 1.3.2 on reads from all samples [39]. To obtain the full sequence of genes split over non-contiguous genomic regions, we merged the Ensembl, Cufflinks and Abyss W transcripts using CAP3 [54]. This was achieved using the novel approach of scaffolding the transcripts using the sequence of their Z gametologues, as detailed in the Supplementary Methods (Additional file 1). Our *de-novo *assembled W gene sequences and their Z gametologue sequences are provided as Additional files 7 and 8.

### Preparation of chromosomes and fluorescent *in-situ *hybridization (FISH) of BAC clones

Mitotic metaphase chromosomes and interphase preparations were generated from the established chicken embryonic fibroblast cell lines. Fluorescent *in-situ *hybridization was carried out as described in the Supplementary Methods (Additional file 1).

### Quantitative reverse transcription-PCR (qRT-PCR)

Dissected stage 1-4 blastoderms were pooled according to sex, including three biological replicates, RNA was extracted and reverse transcribed as previously described [55]. Quantitative real-time PCR was carried out using the Roche UPL Assay Design Center and relative expression was determined using the comparative CT method (ΔΔCT), with samples normalized against *HPRT *and expressed as fold change, as described in the Supplementary methods (Additional file 1).

## Abbreviations

DE: differential expression; FDR: false discovery rate; FISH: fluorescent *in situ *hybridization; FPKM: fragments per kilobase of exon per million fragments mapped; GLM: generalised linear modelling framework; HH: Hamburger and Hamilton; MHM: male hypermethylated; qRT-PCR: quantitative reverse transcription-PCR; RNA-seq: next-generation sequencing of RNA; SPF: specific pathogen free.

## Competing interests

The authors declare that they have no competing interests.

## Authors' contributions

KA, AO, CS and AS designed the RNA seq screen. KA and CS dissected and extracted RNA. ND carried out bioinformatics analysis under guidance of AO. KA carried out lab validation and confirmation of RNA seq results and helped interpret data. KR assisted with lab work. DD and FG carried out FISH analysis. KA, CS, ND and AS wrote the manuscript. All authors read and approved the final manuscript.

## Supplementary Material

Additional file 1 **Supplementary figures (1 to 10), supplementary methods, figure legends for supplementary figures and tables**. Click here for file

Additional file 2 **Table S1a**. Dimorphically expressed Ensembl annotated genes in the Blastoderm. Click here for file

Additional file 3 **Table S1b**. Dimorphically expressed Ensembl annotated genes in the E4.5 gonads. Click here for file

Additional file 4 **Table S1c**. Two tests for dimorphic expression. Click here for file

Additional file 5 **Table S2**. Genes previously implicated in sexual reproduction or gonadogenesis in a variety of models. Click here for file

Additional file 6 **Table S3**. Ensembl Identifiers for W-linked genes and their Z gametologues. Click here for file

Additional file 7 W gene *de-novo *assembly sequences (fasta file). Click here for file

Additional file 8 Z gene *de-novo *assembly sequences (fasta file). Click here for file
