# Supplementary material for: RNA sequencing reveals sexually dimorphic gene expression before gonadal differentiation in chicken and allows comprehensive annotation of the W-chromosome
Source: Genome Biol. 2013 Mar 25;14(3):R26. doi: 10.1186/gb-2013-14-3-r26 (PMC4053838; doi:10.1186/gb-2013-14-3-r26)
Supplement: Additional file 1 — Supplementary figures (1 to 10), supplementary methods, figure legends for supplementary figures and tables. [file gb-2013-14-3-r26-S1.DOCX]

**Supplementary Material for Ayers et al.**

**RNA sequencing reveals sexually dimorphic gene expression before gonadal differentiation in chicken embryos and allows comprehensive annotation of W-chromosome genes.**

Katie L. Ayers^1,5,6*^, Nadia M. Davidson^1,*^, Diana Demiyah^3^, Kelly N. Roeszler^1^, Frank Grützner^4^, Andrew H. Sinclair^1,2,5^, Alicia Oshlack^1^ and Craig A. Smith^1,2,5^.

^1^ Murdoch Childrens Research Institute, Royal Childrens Hospital, Flemington Road, Parkville 3054 Melbourne, VIC, Australia; ^2^ Department of Paediatrics, The University of Melbourne, Flemington Road, Parkville, 3054, Melbourne, VIC, Australia; ^3^ Institute of Biological Sciences, Faculty of Science, University of Malaya, 50603, Kuala Lumpur, Malaysia; ^4^ The Robinson Institute, School of Molecular and Biomedical Science, University of Adelaide, Adelaide, 5005 S.A., Australia; ^5^ Poultry Cooperative Research Centre, Armidale, N.S.W., Australia. ^6^ Department of Genetics, The University of Melbourne, Parkville, 3054, Melbourne, VIC, Australia.

* These authors contributed equally.

Corresponding authors: [craig.smith@mcri.edu.au](mailto:craig.smith@mcri.edu.au), alicia.oshlack@mcri.edu.au

**Supplementary Methods Pages 2 - 10**

**Supplementary Figures Pages 12 – 28**

**Supplementary Table Legends Page 29**

**Supplementary Methods**

**Data**

The RNA libraries consisted of eight samples: two female and two male samples at each time point, where each sample was pooled from either twelve blastoderms or sixteen gonads. The RNA was sequenced at the Australian Genome Research Facility (AGRF) on four lanes of the Illumina HiSeq2000, with all samples run on each lane. This gave a total yield of around 700 million, 100bp read-pairs, which were distributed between samples as: 82.3 and 87.2 million for each of the male blastoderm samples; 76.5 and 82.3 million for each of the female blastoderm samples; 107.2 and 99.7 million for the male gonad samples; and 78.2 and 83.1 million for the female gonad samples. The average fragment length was approximately 160bp with a standard deviation of 110bp so many of the paired-end reads overlapped.

## RNA-seq analysis and differential expression analysis

Analysis of the data followed the three methods shown in Supplementary Figure 8 and described in detail in the Supplementary Methods below [[1-3](#_ENREF_1)].

**Quality Controls**

We used FastQC (Andrews, S 2010 FastQC. http://www.bioinformatics.bbsrc.ac.uk/projects/fastqc)

to check the quality of our dataset. Each read was trimmed 10 bases from either end to remove observed contamination from sample primers at the beginning [[4](#_ENREF_4)] and to remove poor quality bases from the end of each read. We found that a fraction of the reads (5% of the first read and 1% of the second read in each pair) had a Phred quality score with the lowest possible value, two, for all bases in the read. We removed these reads and their pair. Following this cleaning, the Phred quality scores were high, with a median value above 34 for all bases of the reads.

While undertaking the data analysis we found that one of the pooled male blastoderm samples (replicate 2) showed levels of gene expression at 7.4% of the average blastoderm females’ expression in W-linked genes where the other male samples showed no expression at all. We believe this was due to one out of the twelve pooled individuals being a female contaminant in that sample. In the differential expression analysis we handled the contaminated sample by excluding it when estimating tagwise dispersion, but still included it when performing the actual differential expression test in EdgeR [[5](#_ENREF_5)]. For all other results comparing male to female expression provided in the paper we excluded this sample.

**Ensembl Analysis**

**Mapping Reads**

Read pairs were mapped to the chicken genome using Tophat 1.3.1 [[6](#_ENREF_6)] We used the galGal3 version of the chicken genome [[7](#_ENREF_7)] for the Ensembl analysis because a complete annotation was unavailable for galGal4 at the time of writing. Between 80%-83% of read pairs mapped depending on the sample and approximately 86% of those where classified as “properly paired” by Tophat. We then counted the number of reads overlapping Ensembl transcripts, gallusGallus release 67 (Ensembl 2012), using “bedtools multicov” [[8](#_ENREF_8)] with the parameters “-p and –D” which allowed for duplicate reads and required “properly paired” reads. For each gene, we selected the transcript with the highest total counts (counts summed across all samples) to be representative of that gene. Approximately 70% of “properly paired” mapped reads overlapped an Ensembl gene.

**Differential Expression Analysis**

EdgeR was used to analyse the count data for differential expression. We use tagwise dispersion with a smoothing parameter (prior n=1). To ensure that the contaminated male blastoderm replicate did not bias the dispersion estimate, we excluded the male blastoderm samples from the tagwise dispersion estimate and used the other three replica sets: blastoderm female, gonadal female and gonadal male. Because this approach did not account for cases of extreme variation between the two male blastoderm replicates, we rejected any differentially expressed, male blastoderm up-regulated genes when either of the male blastoderm replicate counts accounted for more than 90% of the total blastoderm counts. When performing the statistical test, the mean expression in each group was calculated using all samples including the contaminated one.

EdgeR fits the count data to a generalized linear model and performs hypothesis testing using a likelihood ratio test. We modeled the data using a factorial design with the factors being: tissue/time-point (blastoderm or gonads) and sex (male or female). The design matrix included a coefficient for each combination of factors: male blastoderm (*C_MB_*), female blastoderm (*C_FB_*), male gonads (*C_MG_*) and female gonads (*C_FG_*). The null hypothesis used for the differential expression test between sexes was that the coefficient for male blastoderms was equal to the coefficient for female blastoderms, *C_MB_=C_FB_*, and similarly for the gonads, *C_GM_=C_GF_*. To test for different patterns of differential expression across the time-points we tested for *C_MB_ – C_FB_ = C_MG_ – C_FG_* (different differential expression)_,_ and *C_MB_ + C_FB_ = C_MG_ +C_FG_* (the average difference between tissue). In both of these later tests, we only reported the significant genes that were also differentially expressed between sexes in either the blastoderms or gonads. P-values were adjusted for the false discovery rate using the Benjamini and Hochberg method [[9](#_ENREF_9)]. We called genes with an adjusted p-value below 0.05 as differentially expressed.

**Identification of W Genes**

We identified candidate W genes from amongst the Ensembl genes by filtering for:

- any gene located on the W or W_random chromosomes,
- any gene which was differentially expressed with a female to male fold change of two or greater at both the blastoderm and gonadal time points,
- any gene which was differentially expressed at either time point and had a Z gametologue. Gametologues were identified using BLAT [[10](#_ENREF_10)] to align the untranslated W sequences against Ensembl Z-linked transcripts. A minimum blat score of 200 was required.

We allowed some male counts for these genes because we found that regions within some W-linked genes had high DNA homology with their Z-linked gametologue and therefore some of their reads mapped to the W gene (Supplementary Figure 9).

**Cufflinks Analysis**

We re-mapped our RNA-seq reads to the most recent version of the chicken genome (galGal4) using Tophat and performed genome guided de-novo transcriptome assembly using Cufflinks [[3](#_ENREF_3)]. We followed the same method as above for i) counting reads which overlapping Cufflink genes, ii) analyzing differential expression and iii) identifying W-link candidate genes, as was done for the Ensembl analysis. The total number of candidate W-linked Cufflinks loci after this process was 769. The majority of these loci were thought to be retroviral or pseudo-genes based on their sequence similarity to a known retrovirus, their lack of an intron-exon structure or because they appeared to be a degraded partial version of another gene. We prioritized interesting candidates using a scoring based system. One point was allocated if the gene had:

- three or more exons
- an open reading frame which matched a known protein with > 90% identity over 20 amino acids. We excluded all open reading frames with any similarity to retroviral elements (using BLAST [[11](#_ENREF_11)] or with more than 20% sequence similarity to a repeat (using RepeatMasker, score>255) [[12](#_ENREF_12)].
- an average female fragments per kilobase of exon per million reads mapped (FPKM) greater than two (equivalent to about 40x average coverage)

25, 34, 134 and 576 genes had a score of 3, 2, 1 and 0 respectively. We short-listed all gene with a score of two or greater as candidate W genes.

***de novo* W transcriptome assembly using Abyss**

A *de novo* transcriptome assembly was performed using Abyss version 1.3.2 on reads from all samples [[1](#_ENREF_1)]. The total number of read pairs used was approximately 650 million. We followed the methodology given in [2] by performing the assembly using a range of k-mer lengths, since different values of the k-mer parameter are optimal for different expression levels. We used values of 42, 49, 54, 59, 64, 69, 74 and 79. This yielded an assembly with approximately 17.5, 11.7, 9.7, 7.9, 6, 4.1, 2.2, 0.5 million contigs respectively. The assemblies for each k-mer length were merged and redundant contigs were removed using in-house software. Trans-abyss was not used to merge the assemblies because it was incompatible with the version of Abyss we used at the time the work was done. We then filtered all contigs with fewer than 100 bases and performed scaffolding using SSPACE [[13](#_ENREF_13)]. This reduced the total number of contigs from over 30 million to 2.5 million.

We assessed the performance of the *de novo* assembly against a reference set of transcripts. The chicken genome annotation from RefSeq, comprising over five thousand genes, was used as a reference set as it has been well curated compared to other annotations. The Abyss *de novo* assembly performed poorly for highly expressed genes. We presumed this was due to errors in the reads. Stricter cleaning on the data could have removed these errors, giving better results for highly expressed transcripts, but at the expense of missing low expressed transcripts. For gene discovery, the later is more important, so we chose not to do this. Based on these results and the unrealistically large number of contigs, we did not perform the differential expression and W-linked gene discovery on the de-novo transcriptome assembly from Abyss. However, as the abyss transcripts crossed gaps in the genome, they were useful for extending (or assembling) Ensembl and Cufflinks transcripts across regions of the genome and we used the Abyss de-novo assembly for this purpose instead.

**Merging Gene Sequences**

During both the Ensembl and Cufflinks analyses we noted that in twelve cases, multiple genes coded for the same single protein. While in some cases, such as *HINT-W* and *FAF*, this was due to a copy number greater than one, we found that in most cases there was a single copy of the gene, but it had been split across non-contiguous or gapped regions of the genome. We re-assembled these loci by utilizing the transcripts from our abyss de-novo assembly. By doing so, we were also able to extend/supplement transcripts with sequences which were entirely absent from the genome, as was the case for genes such as *GOLPH3-W* and *SMAD7B-W*. The procedure for merging these sequences is described below, with results presented in Figure 2 and Supplementary Figure 4. This process also allowed us to combine the Ensembl and Cufflinks W candidate gene lists together.

**1. Grouping Transcript from a Single Gene**

We grouped and ordered transcripts that were common to a single gene using the novel approach of scaffolding the transcripts using the sequence of their Z-linked gametologue. This initially involved identifying the gametologues; we aligned our W-linked candidate genes from both the Ensembl and Cufflinks analyses against Ensembl transcripts from the chicken Z chromosome. This was performed using BLAT with a minimum score of 100 and a minimum identity of 85%. For genes in which no Z-linked Ensembl transcript was found, such as *BTF3*, we searched the Z chromosome sequence for a gametologue that was unannotated in the genome.

We then used these gametologue sequences to identify fragments of W genes. We used BLAT a second time, with the same option used previously, to align the gametologues against all our Cufflinks, Ensembl and Abyss transcripts. We did not restrict the alignment to W candidate genes only because some true W-linked transcripts did not pass the filter and scoring criteria described previously. The expected position and orientation of the transcripts within the full gene was taken from the BLAT results.

**3. Assembling Contigs**

We post-processed the BLAT table within the R programming framework prior to assembling the contigs with CAP3 [[14](#_ENREF_14)]: We excluded any transcript with a DNA match of 97% or greater (matches/mismatches+matches) with the gametologue sequences in order to remove transcripts from the Z gene we used to scaffold. We trimmed the ends of transcripts when they had no homology to the gametologue. This was necessary as we found that Cufflinks often extend the transcript sequence beyond the first/final exon for fragmented genes. For transcript with the smallest or largest location along the gene (i.e. those at the ends) no trimming was performed.

The sequences were then ordered by their expected position in the gene and a CAP3 constraint file was created based on this order. We allowed the distance to float between 0 and 10000 bases. This helped to link the sequences and ensured that the ordering was preserved. Finally CAP3 was run and reported the consensus sequence. Initially we performed the steps above for each gene and used all the transcripts available. The assembled contigs were then verified. In some cases, it was necessary to repeat the procedure above by manually selecting the transcripts to go into the CAP3 assembly. When more than one viable transcript was reconstructed, we choose the transcript with the largest open reading frame.

**4. Validation of the Full Sequence**

We validated the sequences in a number of ways:

1. Searching for an open reading frame and using BLAST to compare the length and protein to the chicken Z gametologue and to similar genes in other species. This helped to ensure that we had identified the full open reading frame.
2. Mapping the read pairs back to the assembled transcript to look for: *i)* uniformity of coverage across the transcript; *ii)* reads which overlapped contig boundaries; *iii)* read pairs with a read on either side of the boundary (Figure 2, Supplementary Figure 4); and *vi)* female specific expression. Note that in performing this test, we found a small number of genes for which the coverage was low (an FPKM < 1). This indicated to us that these sequences had initially had reads incorrectly mapped to them (as TopHat uses a looser mapping criteria). We subsequently removed these genes.
3. Finally, for five genes, the ORFs were cloned and sequenced, and in all cases showed very few changes to the assembled sequences.

**Whole Mount *in situ* Hybridization**

Whole mount *in situ* hybridization was used to verify gonadal expression of some candidate W-linked genes, such as *MIER3-W* and *FAF*. Fertile chicken eggs were incubated at 37.8ºC and tissues were harvested at E4.5 or E5.5 of development. Urogenital systems were dissected from PCR-sexed embryos and subjected to whole mount *in situ* hybridization as described previously [[15](#_ENREF_15), [16](#_ENREF_16)] Digoxygenin-labeled RNA probes were used. For *MIER3-W*, the entire 3’UTR fragment was used as a probe and for *FAF*, the entire cDNA was used. Specific gonadal staining was detected in female but mot male tissues hybridised with antisense probes, but not the sense control probes.

**Calculation of W Transcript Expression**

Following the reconstruction of full W-linked transcript sequences, we mapped reads back to the W gene sequence and calculated expression levels. Reads for all samples were mapped to the W sequences using Bowtie2 [[17](#_ENREF_17)] with the options “-k=1” (a read is mapped to no more than a single location) and “L,-0.1,-0.1” (require stricter matching than the default). The Fragments Per Kilobase per Million mapped (FPKM) was calculated for the female blastoderm and gonad samples using only the open reading frame of the sequences. For the non-coding RNAs we used the entire sequence. In both cases, we excluded the 100 bases closest to the edge of the transcripts where read-pairs have reduced mappability. Because we only mapped reads to forty genes, we estimated the number of million reads that would have mapped to the full chicken transcriptome. These values were taken from the Cufflinks analysis. We found this to be a reasonable estimate because the FPKM values we obtained were comparable to those calculated during the Ensembl and Cufflinks analyses.

**Comparison to Z Gametologues**

The W transcripts were compared to their Z gametologues to screen for both expression differences and sequence divergence. We identified gametologues during the merging of the Cufflinks, Ensembl and Abyss gene fragments (see “Merging Gene Sequences” above). In a number of cases we found the Z transcripts to be incomplete or the isoform observed in our data was not present in the Ensembl annotation. We therefore repeated the merging procedure described above for some of the Z genes, this time selecting only those Ensembl, Cufflink and Abyss transcript fragments that matched a Z gametologue with greater than 97% identity. To compare the sequence identity of the W and Z transcripts, we used BLAST. The identity was calculated as the number of matching bases (or amino acids) over the length of the W sequence. Sequence dN/dS was calculated using the seqinR [[18](#_ENREF_18)] function “kaks” which implements the Li estimation method [[19](#_ENREF_19)]. We only calculated dN/dS for non-gapped alignment regions of the amino acid sequence.

The expression levels for the Z transcripts were calculated in the same manner as described above for the W transcripts. For many of our W transcripts, some reads were found to map from the male samples due to high homology between the W and Z gene (Supplementary Figure 9). These counts from the male samples were therefore reassigned to the Z gametologues prior to calculating the FPKM. This adjustment was never greater than 3% of the original Z transcript expression.

The differential expression analysis of W plus Z gene counts in females to Z gene counts in males was performed using edgeR. We adjusted the counts to compensate for the difference in length between the W and Z transcript by scaling the counts in the longer transcript down:

*c^’^_w_* = min(*l_w_,l_z_*)*/l_w_ * c_w_*

*c^’^z =* min(*l_w_,l_z_*)*/l_z_ * c_z_*

Where *c*, *c^’^* were the old and new counts respectively and *l* was the length over which the reads were counted. This resulted in a conservative estimate of p-values. No adjustments were made for %GC or other biases. We expect that these effects should be small given the high similarity of the sequences at the DNA level (with the exception of *HINT*). Differential expression was calculated for total (Z+W) counts for female blastoderms against male blastoderm (Figure 4A) and female gonads against male gonads (Figure 4B).

**Quantitative reverse transcription-PCR (qRT-PCR)**

Dissected stage 1-4 blastoderms were pooled according to sex, including three biological replicates, RNA was extracted and reverse transcribed. Quantitative real time PCR was carried out using the Roche UPL Assay Design Center (https://www.roche-applied-science.com). RT negative samples were included in all assays. Probes were as follows: *KCMF1-W* probe 78 with Forward primer 5’-AGGGGCTCAGTGTGTAAGGA-3’ and Reverse primer 5’-TCCACCGGACTGTTCAGG-3’; *MIER3-W* probe 53 with Forward primer 5’-CAGTCCATAAATGAGGAAATGTCA-3’ and Reverse primer

5’-GCTGCACCACAGAATTGTTTT-3’; *RASA1-W* probe 35 with Forward primer

5’-CGAGCACGATATTCTATGGAGA-3’ and Reverse primer

5’-TACATGAAGCTCTTTCTGAAGTATGAT-3’; *ZNF532-W* probe 147 and Forward primer 5’-CCAAATGTTCTGGTGCTCAG-3’ and Reverse primer 5’-GTAGGCTGGTGTGTGGTGTG-3’; *HPRT* probe 38 together with Forward primer 5’-CGCCCTCGACTACAATGAATA-3’ and Reverse primer 5’-CAACTGTGCTTTCATGCTTTG-3’. Analysis was performed using a LightCycler 480 instrument, LC480 master mix and software (Roche). Relative expression was determined using the comparative CT method (ΔΔCT), with samples normalized against *HPRT* and expressed as fold change. In addition, the efficiency of each primer/probe combination was determined and only combinations with high efficiencies were used.

**Preparation of chromosomes and fluorescent in situ hybridization (FISH) of BAC clones**

Mitotic metaphase chromosomes and interphase preparations were generated from the established chicken embryonic fibroblast cell lines. The material was either directly fixed in methanol/acetic acid (3:1) or incubated in 0.075 M KCl M at 37°C as hypotonic treatment and then fixed. Slides were prepared according to standard procedures. BAC clones were obtained from the Children’s Hospital Oakland Research Institute (CHORI, CA, USA) from the chicken BAC library CH261. PCR was used to confirm that the BAC clones harbored the W genes of interest. The W BAC clones were then mapped to metaphase chromosomes to confirm their location, which was assessed using karyotype for Z and macro chromosomes. DNA (1 µg) from the positive BAC clones were directly labeled with spectrum orange or spectrum green (Vysis) using random primers and Klenow polymerase and hybridized by fluorescence in situ hybridization to chicken metaphase and interphase chromosomes under standard conditions. Briefly, the slides were treated with 100 µg/ml RNase A/2 x SSC 37°C for 30 min and with 0.01% pepsin in 10 mM HCl at 37°C for 10 min. After refixing for 10 min in 1 x PBS, 50 mM MgCl2, 1% formaldehyde, the preparations were dehydrated in an ethanol series. Slides were denatured for 2.5 min at 75°C in 70% formamide, 2x SSC, pH 7.0 and again dehydrated. For hybridization of one half slide, 400–500 ng of probe DNA was co-precipitated with 10–20 μg of boiled chicken genomic DNA (as competitor), and 50 µg salmon sperm DNA (as carrier), and re-dissolved in 50% formamide, 10% dextran sulfate, 2x SSC. The hybridization mixture was denatured for 10 min at 80°C. Pre-annealing of repetitive DNA sequences was carried out for 30 min at 37°C. The slides were hybridized overnight in a moist chamber at 37°C. The slides were then washed three times for 5 min in 50% formamide, 2 x SSC at 42°C and once for 5 min in 0.1 x SSC. Chromosomes and cell nuclei were counterstained with 1 μg/ml DAPI in 2°— SSC for 1 min and mounted in 90% glycerol, 0.1 M Tris–HCl, pH 8.0 and 2.3% DABCO. Images were taken with a Zeiss AxioImagerZ.1 epifluorescence microscope equipped with a CCD camera and Zeiss Axiovision software. W chromosome mapping was confirmed by the presence of one signal per cell spread in female but not in male cells.

**References**

1. Simpson JT, Wong K, Jackman SD, Schein JE, Jones SJ, Birol I: **ABySS: a parallel assembler for short read sequence data.** *Genome Res* 2009, **19:**1117-1123.

2. Robertson G, Schein J, Chiu R, Corbett R, Field M, Jackman SD, Mungall K, Lee S, Okada HM, Qian JQ, et al: **De novo assembly and analysis of RNA-seq data.** *Nat Methods* 2010, **7:**909-912.

3. Trapnell C, Roberts A, Goff L, Pertea G, Kim D, Kelley DR, Pimentel H, Salzberg SL, Rinn JL, Pachter L: **Differential gene and transcript expression analysis of RNA-seq experiments with TopHat and Cufflinks.** *Nat Protoc* 2012, **7:**562-578.

4. Hansen KD, Brenner SE, Dudoit S: **Biases in Illumina transcriptome sequencing caused by random hexamer priming.** *Nucleic Acids Res* 2010, **38:**e131.

5. Robinson MD, McCarthy DJ, Smyth GK: **edgeR: a Bioconductor package for differential expression analysis of digital gene expression data.** *Bioinformatics* 2010, **26:**139-140.

6. Trapnell C, Pachter L, Salzberg SL: **TopHat: discovering splice junctions with RNA-Seq.** *Bioinformatics* 2009, **25:**1105-1111.

7. Consortium ICG: **Sequence and comparative analysis of the chicken genome provide unique perspectives on vertebrate evolution.** *Nature* 2004, **432:**695-716.

8. Quinlan AR, Hall IM: **BEDTools: a flexible suite of utilities for comparing genomic features.** *Bioinformatics* 2010, **26:**841-842.

9. Benjamini Y, and Hochberg, Y. : **Controlling the false discovery rate: a practical and powerful approach to multiple testing. .** *Journal of the Royal Statistical Society Series B* 1995, **57:**289–300.

10. Kent WJ: **BLAT--the BLAST-like alignment tool.** *Genome Res* 2002, **12:**656-664.

11. McGinnis S aMT: **BLAST: at the core of a powerful and diverse set of sequence analysis tools.** *Nucleic Acids Research* 2004, **32**.

12. Tempel S: **Using and understanding RepeatMasker.** *Methods Mol Biol* 2012, **859:**29-51.

13. Boetzer M, Henkel CV, Jansen HJ, Butler D, Pirovano W: **Scaffolding pre-assembled contigs using SSPACE.** *Bioinformatics* 2011, **27:**578-579.

14. Huang X, Madan A: **CAP3: A DNA sequence assembly program.** *Genome Res* 1999, **9:**868-877.

15. Andrews JE, Smith CA, Sinclair AH: **Sites of estrogen receptor and aromatase expression in the chicken embryo.** *Gen Comp Endocrinol* 1997, **108:**182-190.

16. Smith CA, Andrews JE, Sinclair AH: **Gonadal sex differentiation in chicken embryos: expression of estrogen receptor and aromatase genes.** *J Steroid Biochem Mol Biol* 1997, **60:**295-302.

17. Langmead B, Salzberg SL: **Fast gapped-read alignment with Bowtie 2.** *Nat Methods* 2012, **9:**357-359.

18. Charif D, Thioulouse J, Lobry JR, Perriere G: **Online synonymous codon usage analyses with the ade4 and seqinR packages.** *Bioinformatics* 2005, **21:**545-547.

19. Li WH: **Unbiased estimation of the rates of synonymous and nonsynonymous substitution.** *J Mol Evol* 1993, **36:**96-99.

20. Dennis G, Jr., Sherman BT, Hosack DA, Yang J, Gao W, Lane HC, Lempicki RA: **DAVID: Database for Annotation, Visualization, and Integrated Discovery.** *Genome Biol* 2003, **4:**P3.

**Supplementary Figures**

**Supplementary Figure 1. Validation of RNA-seq**

Expression of known gonadal-related genes in day 4.5 embryonic chicken gonads (log scale), based on RNA-seq. *AMH* and *DMRT* are male biased while *FOXL2* is female biased. *SOX9* is not sexually dimorphic and aromatase is not yet transcribed. These results are consistent with the known expression profiles of these genes (see main results for references), validating the RNA seq data. (**, p<0.01)

**
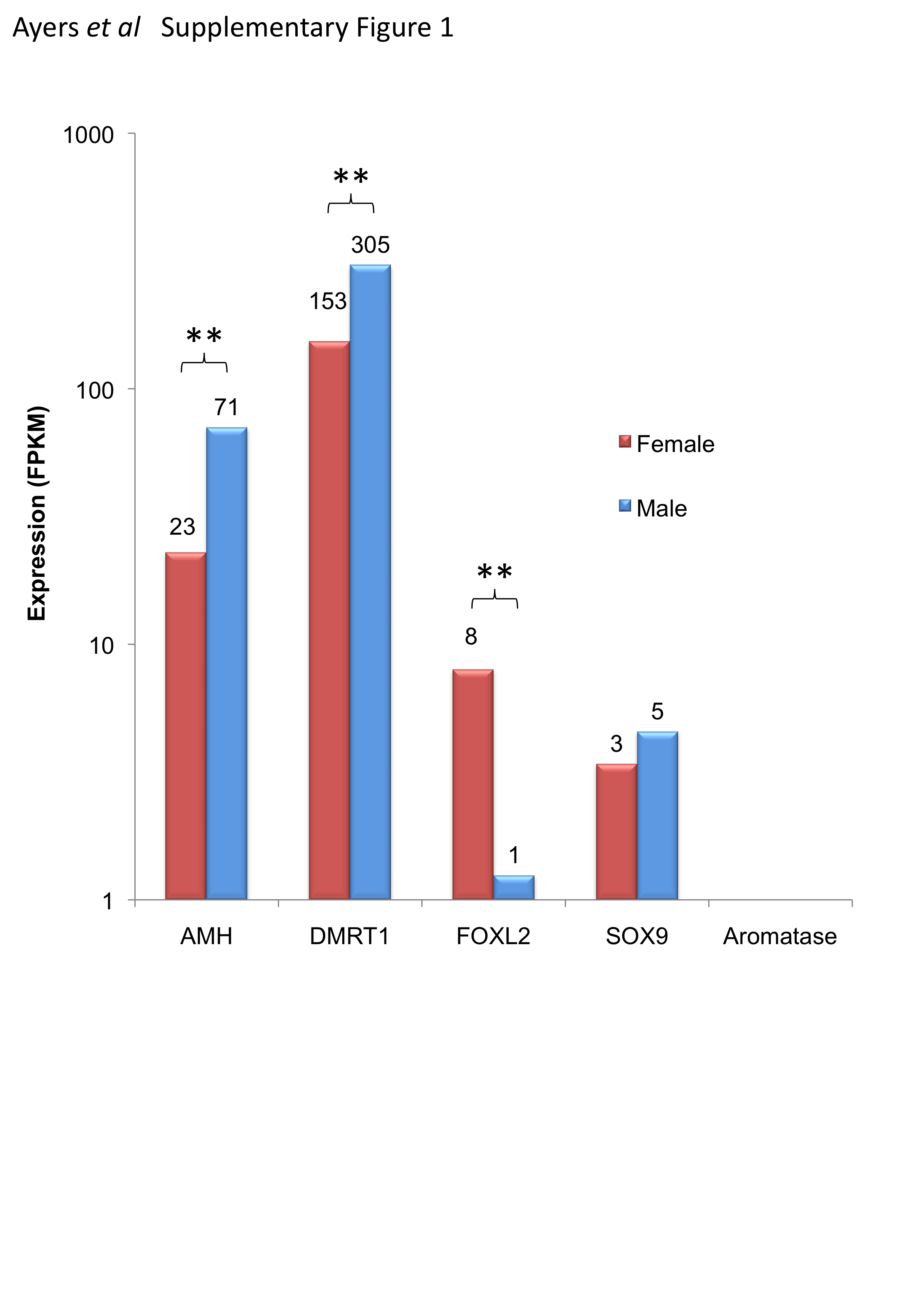
Supplementary Figure 2.** **Male to female expression fold change across the Z chromosome in the blastoderms and gonads.**

The ratio for each Ensembl gene (blue dots) is shown along with a running median of 50 genes (black line). The previously identified Male HyperMethylated (MHM) “valley” is detectable here as a clear decline in the fold change at around 30Mb. However, there is a similar region around 72-80Mb. The median value for all Z genes (grey line) is at approximately 1.6 and confirms partial dosage compensation along the Z chromosome at both time points.

**Supplementary Figure 3. Many mapped reads do not fall within annotated genes**

Mapped read that do not fall in annotated genes. For the autosomes, sex chromosomes and the Un_random (virtual) chromosome, the fraction of reads on the chromosome that map outside annotated genes is shown. Compared to the rest of the chicken genome, the Un_random (“Un”) has a very high percentage of reads that fall outside annotated genes.

**Supplementary Figure 4. Complete Transcript Sequences.**

Complete transcript sequences for all W-expressed genes were determined using a combined approach of assembling transcripts from Cufflinks, the Abyss *de-novo* assembly and the latest chicken annotation data (Ensembl). This figure shows the additional open reading frame or sequence (non-coding genes) for the remaining W-linked genes that are not presented in Figure 2 of the paper. The hatched rectangles represent the different genomic regions to which sequences could be aligned: the W/W_random chromosome (red), the Un_random chromosome (green), autosomes (grey) and gaps represent absent genomic sequence. Note that for some genes multiple genomic regions overlap the same sequence, indicating a copy number greater than one. The coloured bars below show the corresponding transcripts defined by the Ensembl (aqua), Cufflinks (yellow) and ABySS (blue) analyses. The plots along the top represent the read coverage for the female gonadal sample (black) and the blastoderm sample (grey).

**Supplementary Figure 5. Whole mount validation of gene expression in embryonic gonads.**

Whole mount in situ hybridization was used to confirm female expression of some W-linked candidates, complementing the qPCR validation (Figure 3A).

A) *MIER3-W* expression in female (ZW) gonads at E5.5.

B) No *MIER3-W* expression in male (ZZ) gonads.

C) *FAF* expression in E4.5 females (ZW) gonads at E4.5

D) No *FAF* expression in male (ZZ) gonads at E4.5

**
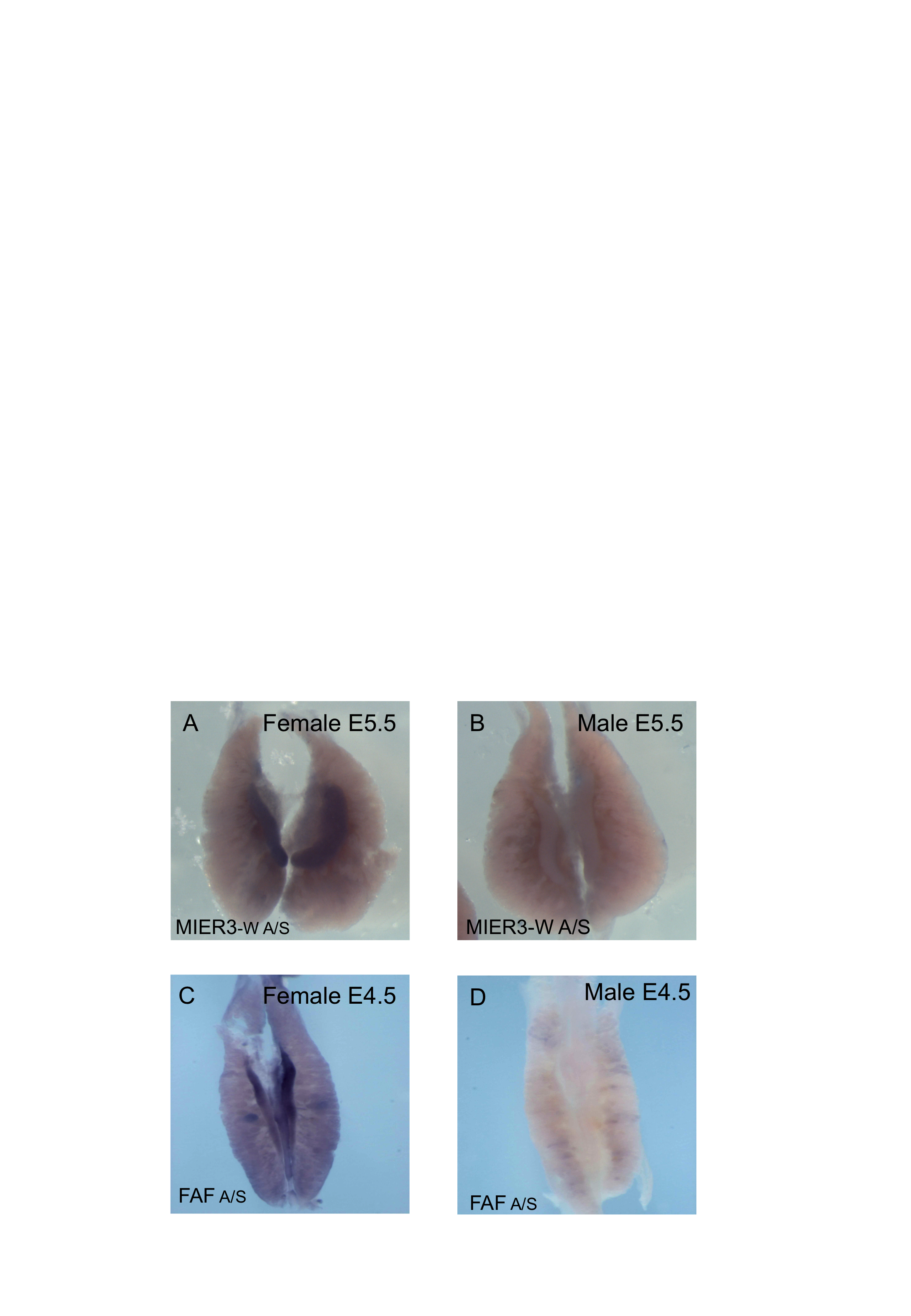
**

**Supplementary Figure 6. Confirmation of W-linkage**

BAC clone FISH analysis continued from Figure 3. Several BAC clones hybridised to chick W chromosomes in metaphase spreads (A-C). Ch261-46G16 hybridised to numerous chromosomes including the Z, Chr1 and microchromosomes, however a strong signal was detected in females (E), that was absent in males (D) representing the W. Genes that are present on each BAC clone are shown in the Table.

**
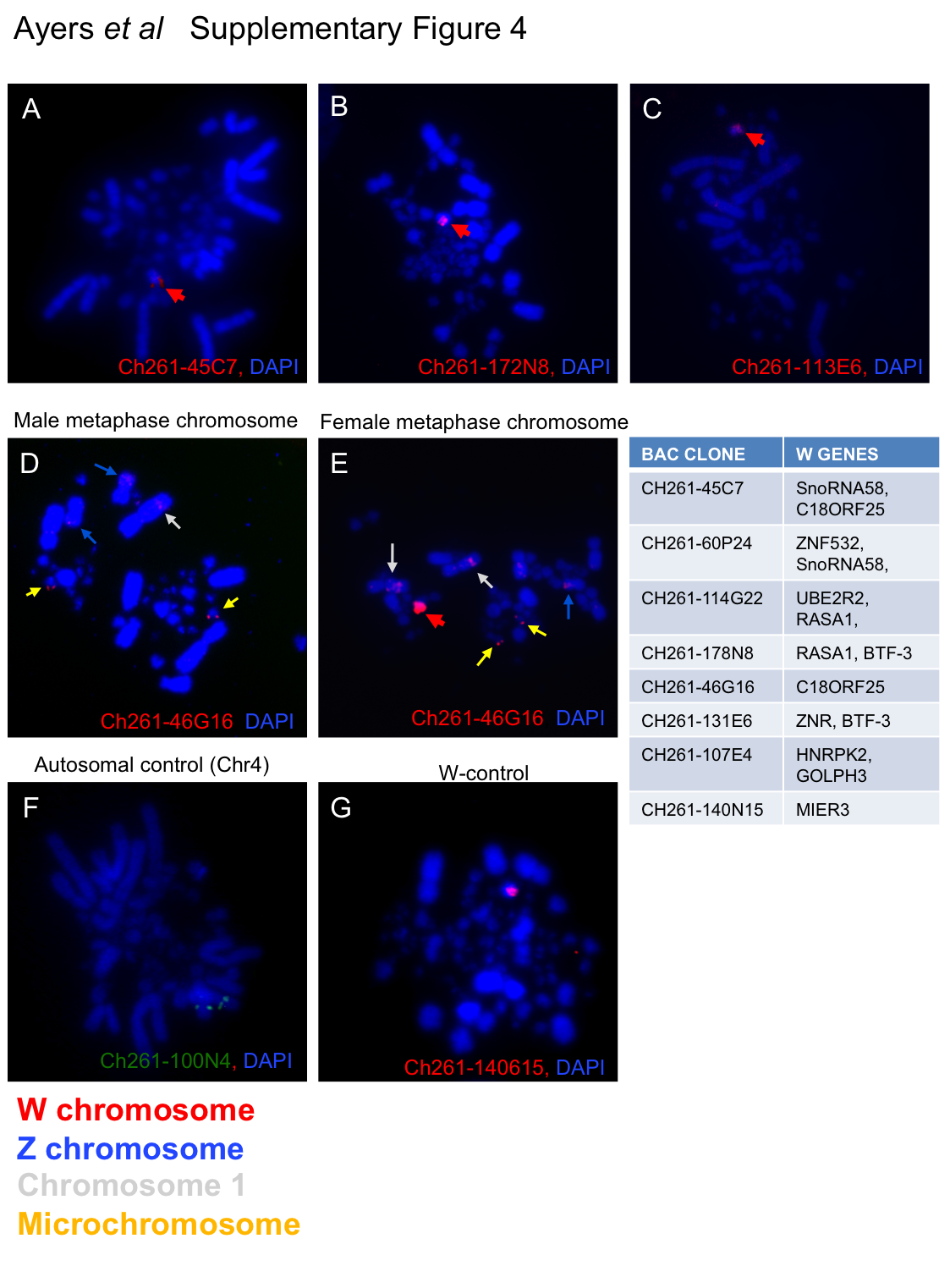
Supplementary Figure 6 continued. Confirmation of W-linkage for potential W genes**


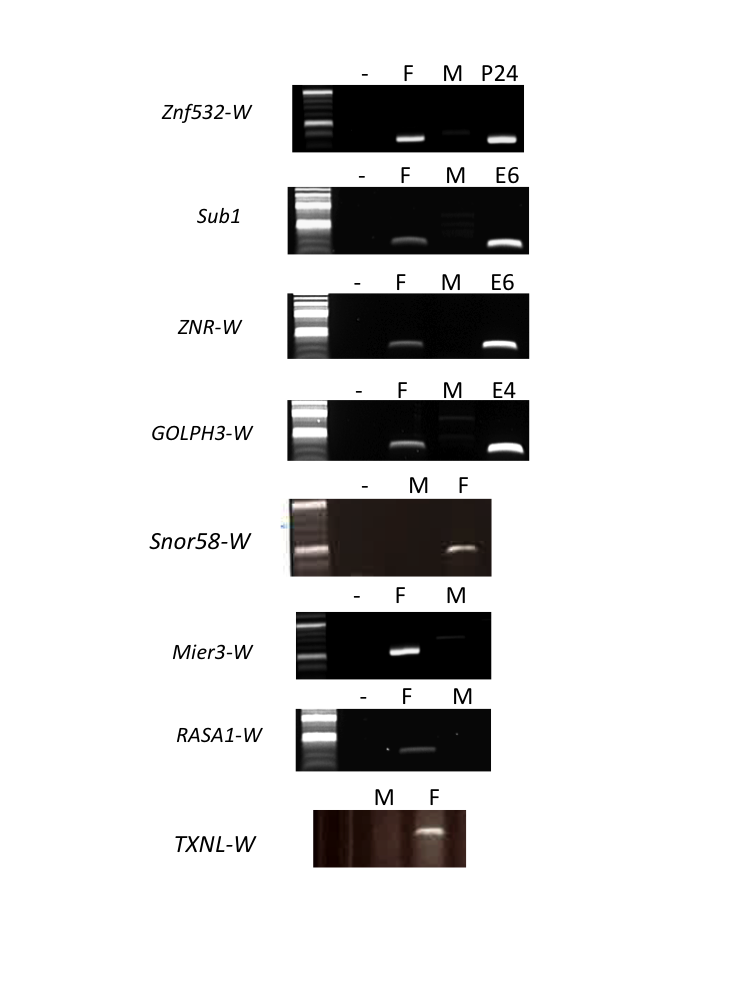
PCR primers and gel pictures for genes amplified off W clone BACs (numbers P24, E6 etc) or from genomic (female – F, male – M) DNA.

| Primer Name | Primer Sequence |
| --- | --- |
| MIER3_W_3UTR_For | GCTGTAGGGGAACTGTTA |
| MIER3_W_3UTR_Rev | CTTCGTTCAACTCTCCCA |
| KA.BTF3-WF | ACGAGCCTTCATCATCAC |
| KA.BTF3-W.R | AAACCAGACCCTATAGCCA |
| Mier3-W.F | CTCTCATGTGGTACTAAACT |
| Mier3-W.R | TCCCAAAACCATGATGCT |
| Znf532-W.F | AAGTTTAGCAGAAGGCAGT |
| Znf532-W.R | ACATGGGAAACACCAACC |
| Rasa-W.F | GCTACTCCGTCCTATGTC |
| Rasa-W.R | TTTTCCTCCACAGTCCTC |
| ZNR-W.F | GCTGCTGCTCAATATAGGT |
| ZNR-W.R | ATTCCCTTCCACTTCCAC |
| Golph3-W.F | CGCTATCTCTGCCTCTCT |
| Golph3-W.R | TGCAATCTCCCTTCTCGT |
| SNOR58-W-F | CTGTGTTTCTGTGCTCCT |
| SNOR58-W-R | ACCAAGCTATGACTAACCAA |
| mir7b-W_F | TTAATCAAGTAGCCCTCC |
| mir7b-W_R | GTTAACATTTGGCAGAGAAG |
| smad7b-w.PCR.F | TACCGAGTTCGTCTGCTG |
| smad7b-w.PCR.R | CCCCTGGCTCCTGAATAA |
| Sub1-W.F | TTGACAAAAAGGCGAAGC |
| Sub1-W.R | ACCTTCTTGATCCATCCA |
| Nedd4.w.f | ATGGGCTGCTTCATAGTT |
| Nedd4.w.R | AGCCACAAATCTCACGTAAA |
| TXNL1.W.F | ATCTGGTGCCGCCTGTGT |
| TXNL1.W.R | CCGCATGGTGAATTTCAC |

**Supplementary Figure 7. dN/dS across each W transcript.**

dN/dS was calculated using a sliding window across the aligned W and Z protein sequences. A window size of 100 amino acids was used with a step size of 5 amino acids. The average dN/dS across each transcript can be found in Table 1, with HINTW showing the least purifying selection of all the W genes.

**
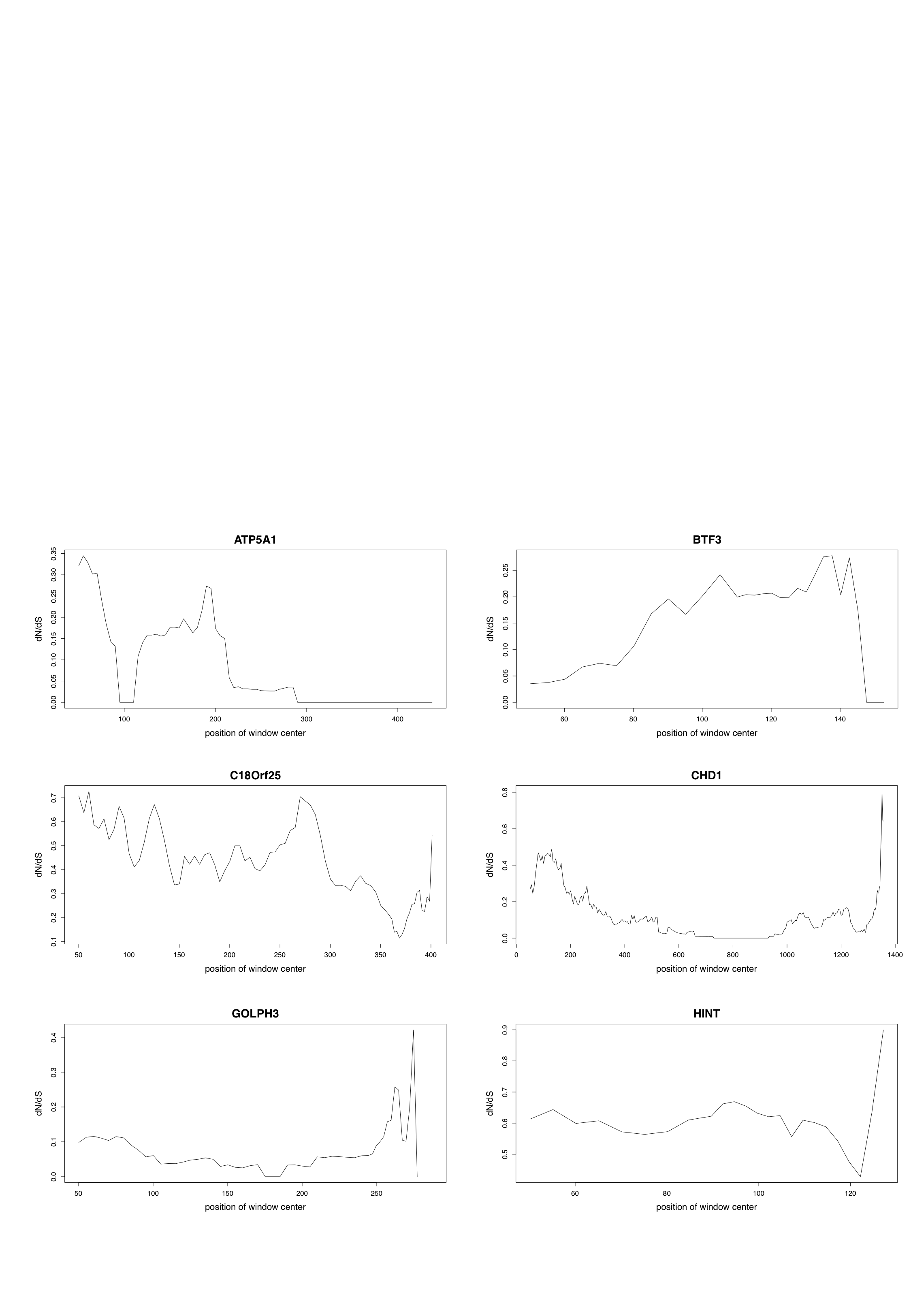
**

**
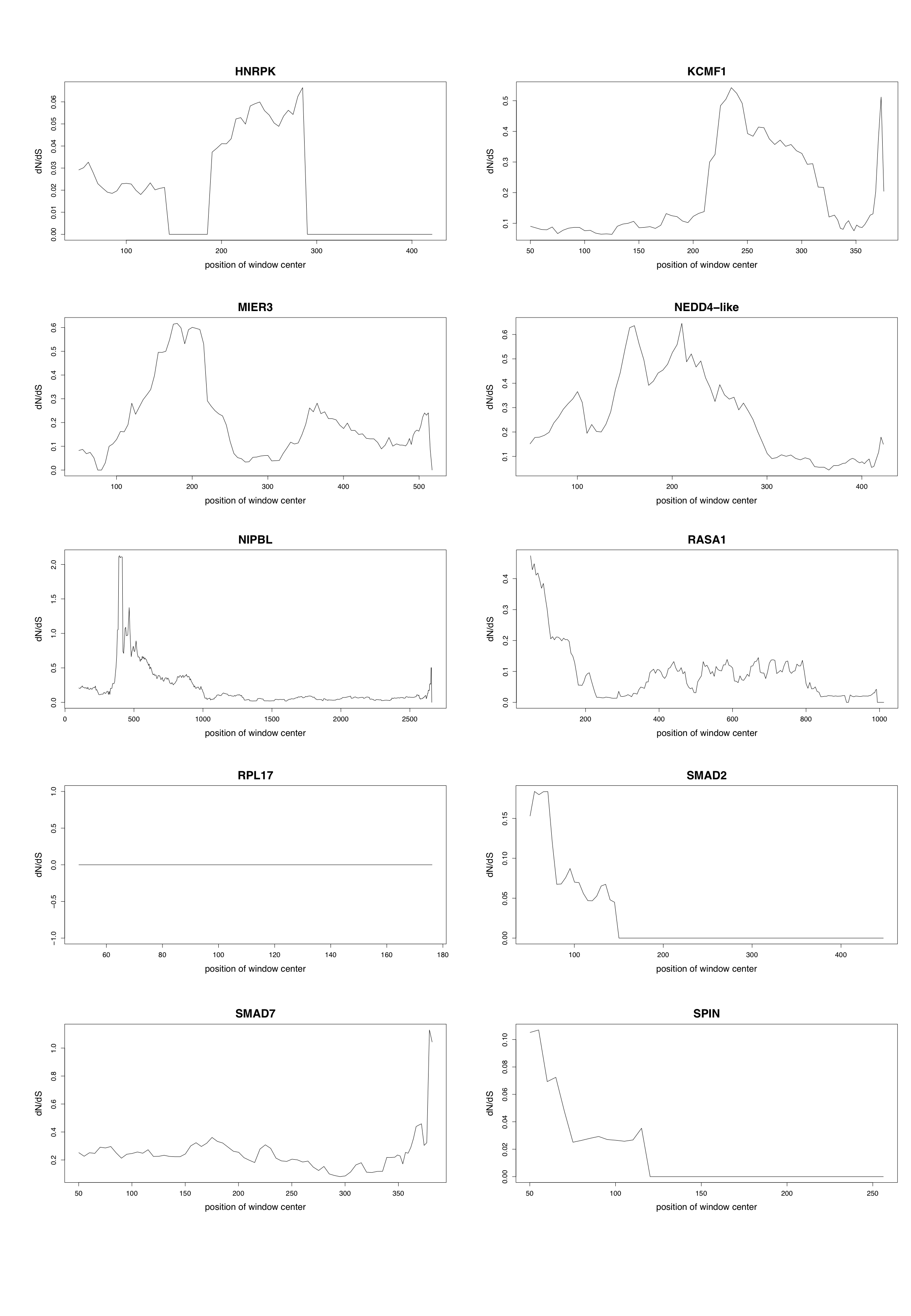
**

**
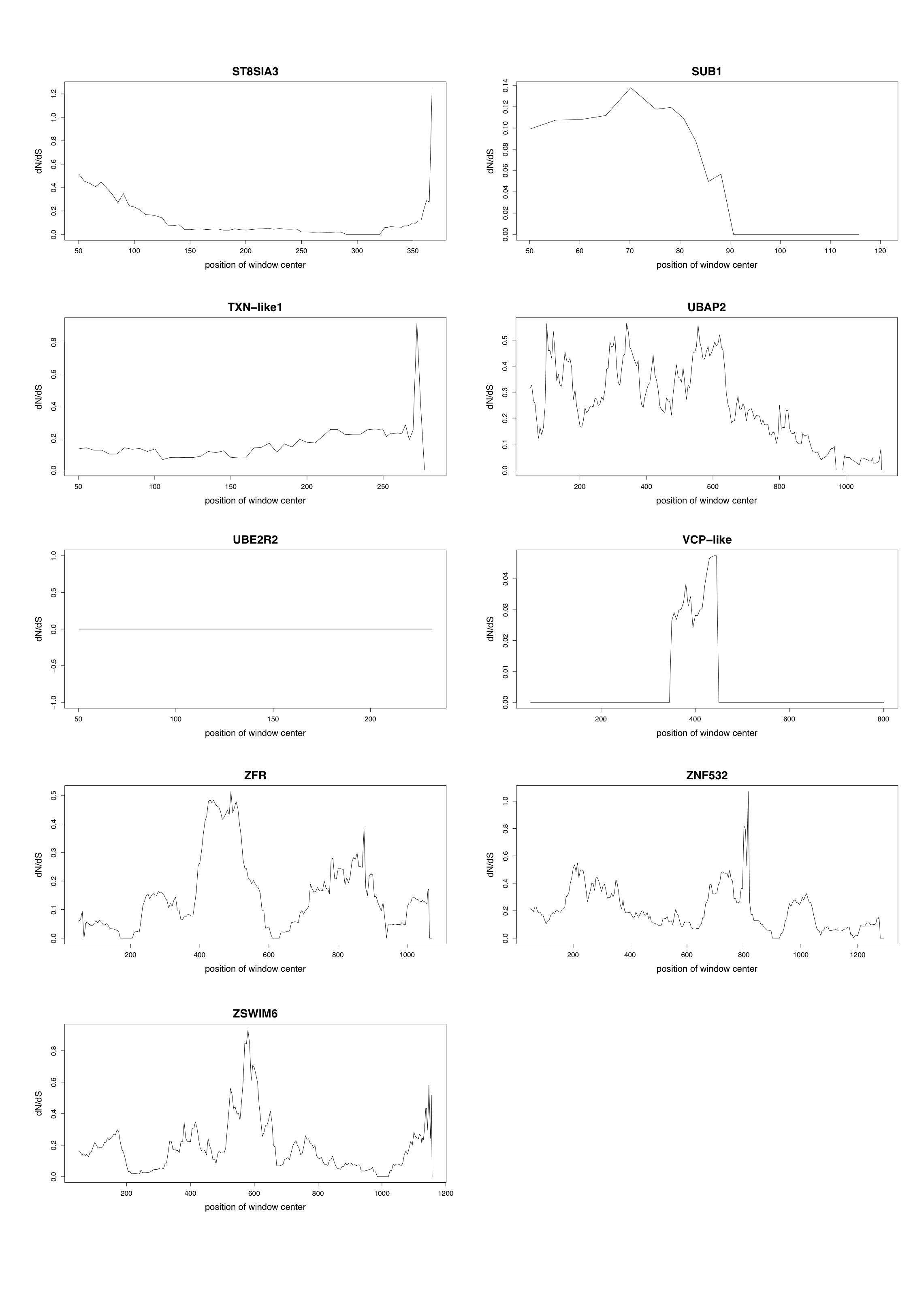
**

**Supplementary Figure 8. Steps in the data analysis workflow.**

Three approached were used to identify the W-linked genes and to reconstruct their full sequence: a count based analysis using the Ensmebl annotation ; a Cufflinks genome guided assembly ; and an Abyss *de novo* transcriptome assembly.

**
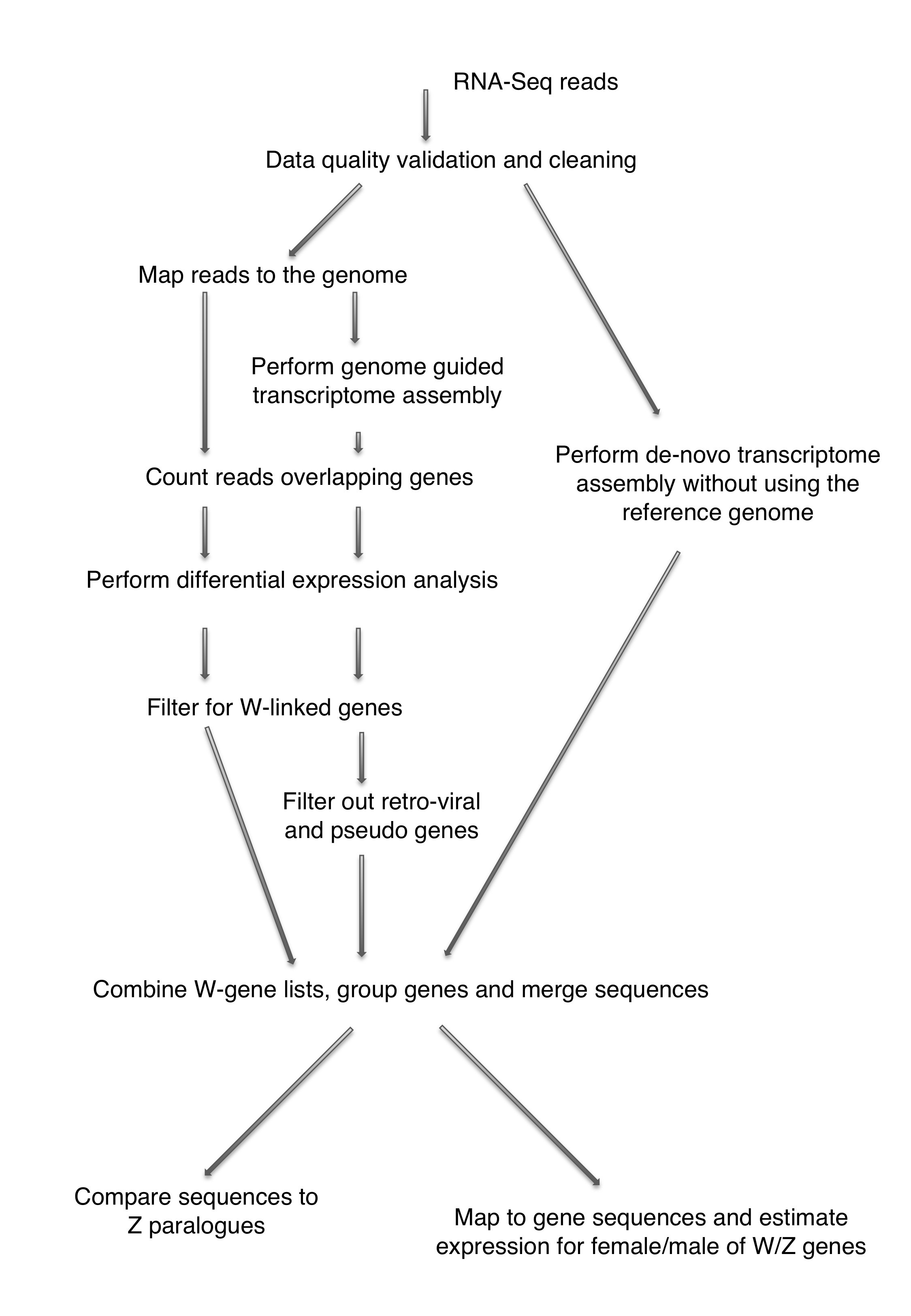
**

**Supplementary Figure 9. False mapping of male reads to the W due to sequence homology**

Some male reads will map to W transcripts due to regions of high homology with their Z gametologues. We show the average read coverage ratio of male samples to female samples (y-axis) along the open reading frame for several W transcripts. Below in colour we show the similarity of the DNA sequence in a sliding window of 80 bases compared to the Z gametologue. 80 bases is the read length after trimming. The six genes shown are those for which the average male to female count ratio was greater than 1% on average.


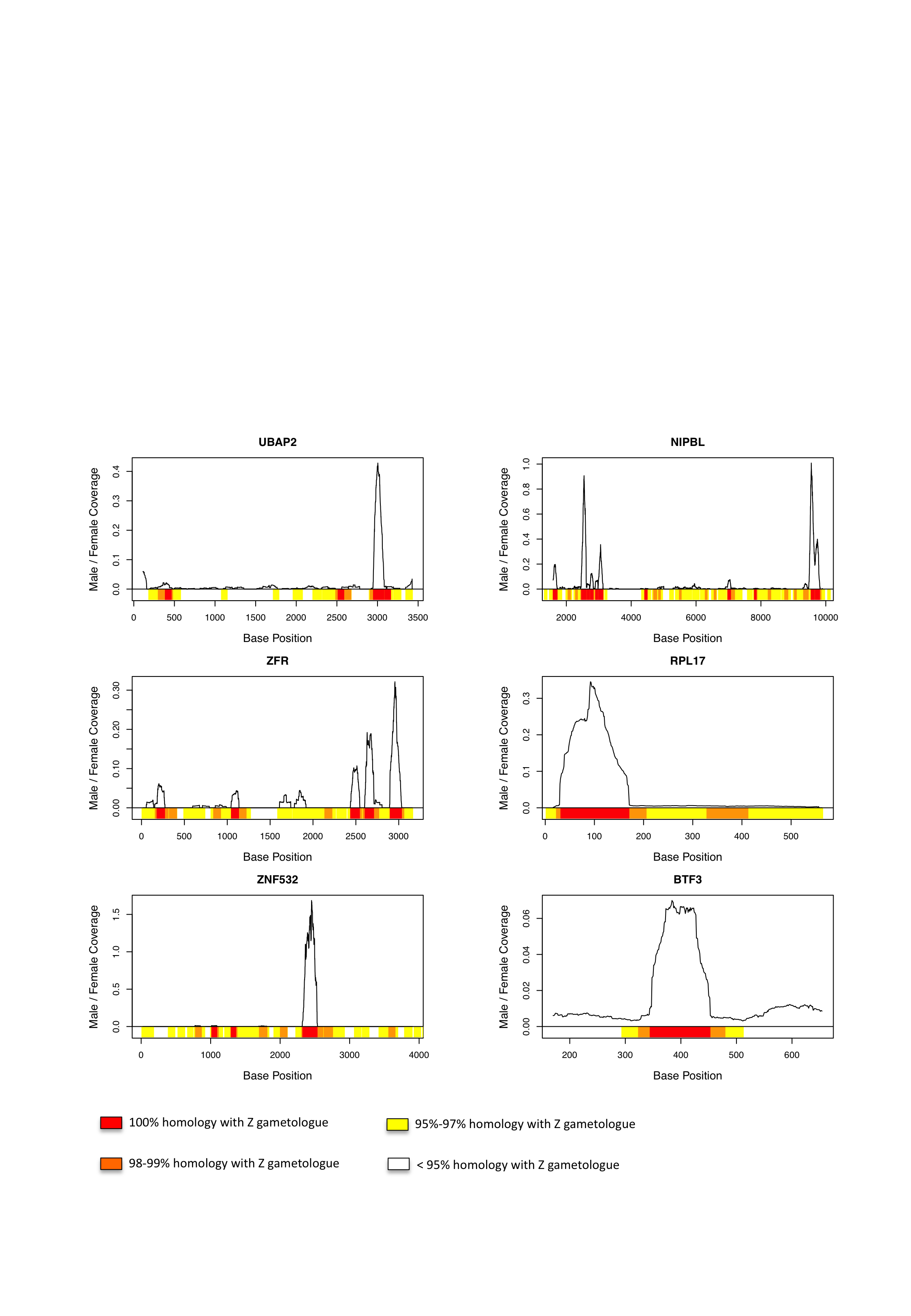


**Supplementary Figure 10. DAVID analysis of differentially expressed genes.**

Genes differentially expressed in either only the blastoderm (A) or only the d4.5 gonads (B) were assessed for enrichment of specific pathways or functions using the DAVID program [[20](#_ENREF_20)]. The Benjamini P-value is corrected for multiple testing.

A


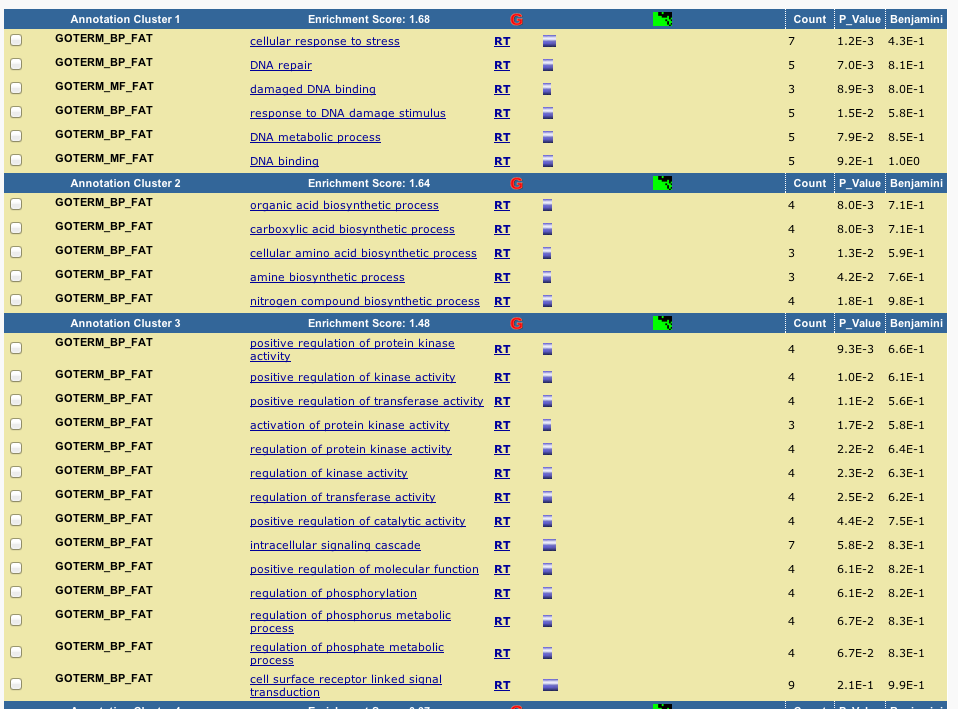


B


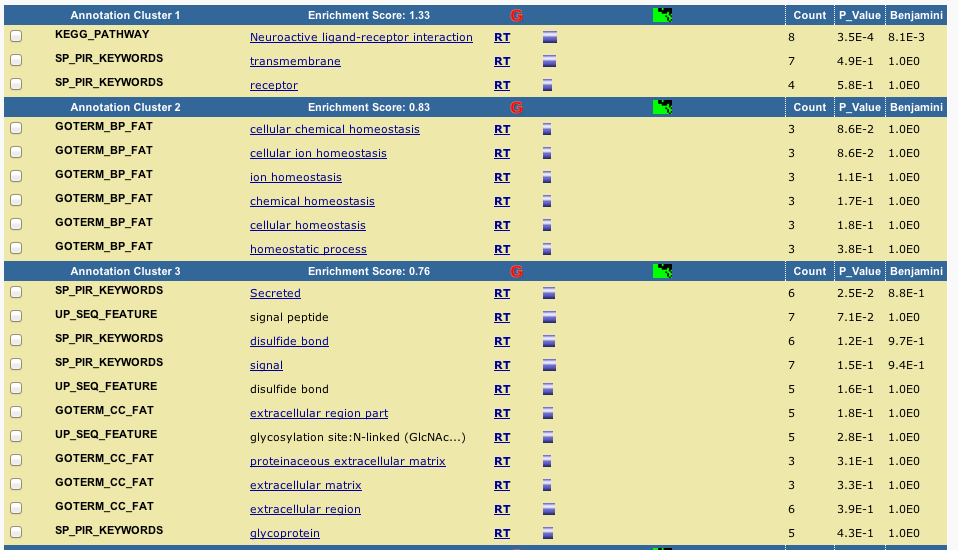


**Table Legends**

**Additional file 2 - Table S1a**

Dimorphically expressed Ensembl annotated genes in the Blastoderm. Ensembl ID numbers, wikigene names, expression in FPKM for male and female, Fold change (Female:Male) and P-Value are shown. Female up-regulated genes are in red, while male up-regulated genes are in blue. In grey are genes currently annotated to the Un_random chromosome or autosomes that we found represent fragments of W genes.

**Additional file 3 - Table S1b**

Dimorphically expressed Ensembl annotated genes in the E4.5 gonads. Ensembl ID numbers, wikigene names, expression in FPKM for male and female, Fold change (Female:Male) and P-Value are shown. Female up-regulated genes are in red, while male up-regulated genes are in blue. In grey are genes currently annotated to the Un_random chromosome or autosomes that we found represent fragments of W genes.

**Additional file 4 - Table S1c**

We tested in two ways for different sexually dimorphic expression between blastoderm and gonad tissue. Firstly, we tested for a difference in the fold change between male:female expression between the blastoderm and gonad (FDR <0.05), i.e. the difference of differences, see Supplementary Methods. Secondly we tested for genes that showed a difference in their total average expression from blastoderms to gonads (FDR <0.05). Because we were only interested in genes involved in sexual development, we only present the sub-set of genes which where both significantly differently expressed in one of the tests above and significantly differentially expressed between females and males at either of the time-points. This data is summarized in Figure 1C and 1D.

**Additional file 5 - Table S2**

Genes previously implicated in sexual reproduction or gonadogenesis in a variety of models.

**Additional file 6 - Table S3**

Ensembl Identifiers for W-linked genes and their Z gametologues, together with the nearest NCBI RefSeq. number.
